# Supplementary material for: Retrotransposon-mediated disruption of a chitin synthase gene confers insect resistance to Bacillus thuringiensis Vip3Aa toxin
Source: PLoS Biol. 2024 Jul 2;22(7):e3002704. doi: 10.1371/journal.pbio.3002704 (PMC11249258; doi:10.1371/journal.pbio.3002704)
Supplement: S1 Table — (DOCX) [file pbio.3002704.s001.docx]

S1 Table. Selection of the Sfru_R3 strain for resistance to Vip3Aa.

| Generation | N^a^ | EC_50_ (95% CI)^b^ | Slope ± SE | Resistance ratio ^c^ | Selection concentration  (μg Vip3Aa per g diet) | Year and month |
| --- | --- | --- | --- | --- | --- | --- |
| 1 | 144 | 0.077 (0.062 - 0.092) | 4.7 ± 0.9 | 1.0^d^ | 2.22 | 2020.06 |
| 3 | 384 | 0.85 (0.30 - 3.67) | 8.4 ± 0.9 | 11.04 | 2.22 | 2020.08 |
| 12 | 384 | 0.57 (0.33 - 0.98) | 8.2 ± 0.9 | 7.40 | 4.44 | 2021.05 |
| 13 | 192 | 16.2 (11.2 - 32.7) | 11 ± 2.6 | 210 | 8.88 | 2021.07 |
| 14 | 192 | > 8^f^ | NA^e^ | > 104 | 8.88 | 2021.08 |
| 15 | 144 | 80^f^ | NA | 1040 | 12.0 | 2021.09 |
| 16 | 144 | > 100^f^ | NA | > 1300 | 24.0 | 2021.11 |
| 17 | 156 | 428 (312 - 617) | 4.4 ± 1.12 | 5560 | 24.0 | 2021.12 |

^a^ Number of neonates tested.
^b^ Median effective concentration (EC_50_); concentration that caused 50% of neonates to die or not develop to the third instar in

7 days and its 95% confidence interval in μg Vip3Aa per cm^2^ diet.

^c^ EC_50_ for a strain or progeny from a cross divided by the EC_50_ for SS

^d^ Data for SS in top row (Generation 1), all other rows report the data for Sfru_R3

^e^ Not available, could not be calculated.

^f^ Response (dead larvae plus larvae that did not develop to third instar) at the highest concentration tested (in μg Vip3Aa per cm^2^ diet): 0% at 8 in generation 14, 50% at 80 in generation 15, and 4% at 100 in generation 16 (n = 24 for each response value).
