# Supplementary material for: Retrotransposon-mediated disruption of a chitin synthase gene confers insect resistance to Bacillus thuringiensis Vip3Aa toxin
Source: PLoS Biol. 2024 Jul 2;22(7):e3002704. doi: 10.1371/journal.pbio.3002704 (PMC11249258; doi:10.1371/journal.pbio.3002704)
Supplement: S2 Table — (DOCX) [file pbio.3002704.s002.docx]

**S2 Table. Survival from first instar to adult eclosion of *S. frugiperda* strains Sfru_R3 and SS on Bt and non-Bt maize.**

| Maize | Strain | Survival (%)^a^ | SE (%) | Relative survival (%) ^b^ |
| --- | --- | --- | --- | --- |
| Non-Bt | SS | 60.7 | 1.0 | NA^c^ |
| Non-Bt | Sfru_R3 | 83.8 | 1.5 | NA^c^ |
| Vip3Aa | SS | 0.0 | 0.0 | 0.0 |
| Vip3Aa | Sfru_R3 | 23.2 | 4.5 | 27.6 |
| Cry1Ab | SS | 1.4 | 0.7 | 2.3 |
| Cry1Ab | Sfru_R3 | 3.7 | 1.4 | 4.4 |
| Cry1Ab + Vip3Aa | SS | 0.0 | 0.0 | 0.0 |
| Cry1Ab + Vip3Aa | Sfru_R3 | 0.0 | 0.0 | 0.0 |

^a^ Mean based on three replicates with 72 larvae per replicate (216 larvae per survival value).

^b^ 100% times survival on Bt maize divided by survival on non-Bt maize for the same strain.
