# Supplementary material for: Retrotransposon-mediated disruption of a chitin synthase gene confers insect resistance to Bacillus thuringiensis Vip3Aa toxin
Source: PLoS Biol. 2024 Jul 2;22(7):e3002704. doi: 10.1371/journal.pbio.3002704 (PMC11249258; doi:10.1371/journal.pbio.3002704)
Supplement: S3 Table — (DOCX) [file pbio.3002704.s003.docx]

| **S3 Table. Responses of strains SS and Sfru_R3 to Cry1Ab, Cry1Ac, Cry1Fa, and Cry2Ab.** | | | | | |  |  |
| --- | --- | --- | --- | --- | --- | --- | --- |
| Bt protein | Strain^a^ | EC_50_ (95% CI)^b^ | Slope ± SE | RR^c^ |  |  |  |
| Cry1Ab | SS | 0.16 (0.089 - 0.22) | 1.8 ± 0.4 | 1.0 |  |  |  |
| Cry1Ab | Sfru_R3 | 0.32 (0.15 - 0.58) | 1.0 ± 0.3 | 2.0 |  |  |  |
| Cry1Ac | SS | 2.95 (1.52 - 17.95) | 1.0 ± 0.3 | 1.0 |  |  |  |
| Cry1Ac | Sfru_R3 | 2.56 (1.53 - 8.22) | 1.2 ± 0.3 | 0.9 |  |  |  |
| Cry1Fa | SS | 0.0071 (0.0057 - 0.010) | 4.0 ± 0.9 | 1.0 |  |  |  |
| Cry1Fa | Sfru_R3 | 0.014 (0.009 - 0.13) | 0.4 ± 0.8 | 2.0 |  |  |  |
| Cry2Ab | SS | 0.094 (0.068 - 0.12) | 2.7 ± 0.5 | 1.0 |  |  |  |
| Cry2Ab | Sfru_R3 | 0.17 (0.12 - 0.23) | 1.8 ± 0.4 | 1.8 |  |  |  |
| ^a^ 144 neonates were tested for each strain and Bt protein. | | |  |  |  |  |  |
| ^b^ Median effective concentration (EC_50_): concentration that caused 50% of neonates to die or fail  to advance to the third instar in 7 days and its 95% confidence interval; in μg Vip3Aa per cm^2^ diet. | | | | | | | |
| ^c^ Resistance ratio: EC_50_ for a strain divided by the EC_50_ for SS. | | | | | | | |
