# Supplementary material for: Retrotransposon-mediated disruption of a chitin synthase gene confers insect resistance to Bacillus thuringiensis Vip3Aa toxin
Source: PLoS Biol. 2024 Jul 2;22(7):e3002704. doi: 10.1371/journal.pbio.3002704 (PMC11249258; doi:10.1371/journal.pbio.3002704)
Supplement: S4 Table — (DOCX) [file pbio.3002704.s004.docx]

**S4 Table. Dominance of Vip3Aa resistance in *Spodoptera frugiperda* based on survival of Sfru_R3, SS, and their F1 progeny.**

| Concentration  (μg Vip3Aa per cm^2^ diet) | Dominance (*h*) |
| --- | --- |
| 0.25 | 0.61 |
| 0.5 | 0.45 |
| 1 | 0.065 |
| 2 | 0.00 |
