# Supplementary material for: Retrotransposon-mediated disruption of a chitin synthase gene confers insect resistance to Bacillus thuringiensis Vip3Aa toxin
Source: PLoS Biol. 2024 Jul 2;22(7):e3002704. doi: 10.1371/journal.pbio.3002704 (PMC11249258; doi:10.1371/journal.pbio.3002704)
Supplement: S5 Table — (DOCX) [file pbio.3002704.s005.docx]

**S5 Table. Observed mortality at 2 μg Vip3Aa per cm^2^ diet versus mortality expected assuming resistance is controlled by a recessive allele at a single locus.**

| Parameter | Pooled backcross^a^ | Pooled F_2_^b^ |
| --- | --- | --- |
| n ^c^ | 768 | 384 |
| Observed dead (O) | 410 | 299 |
| Expected dead (E) ^d^ | 384 | 288 |
| *P* ^e^ | 0.20 | 0.39 |

| Strain or cross | Mortality (%) |
| --- | --- |
|  |  |
| SS | 100.0 ± 0.0 |
| Sfru_R3 | 0.0 ± 0.0 |
| F1a (Sfru_R3♂ × SS♀) | 100 ± 0.0 |
| F1b (Sfru_R3♀ × SS♂) | 100 ± 0.0 |
| Pooled F1 | 100.0 ± 0.0 |
| F2a (F1a × F1a) | 76.0 ± 2.9 |
| F2b (F1b × F1b) | 79.7 ± 3.3 |
| Pooled F2c | 77.9 ± 2.2 |
| BCR1 (Sfru_R3♂ × F1a♀) | 55.2 ± 2.3 |
| BCR2 (Sfru_R3♀ × F1a♂) | 52.6 ± 4.4 |
| BCR3 (Sfru_R3♂ × F1b♀) | 50.0 ± 5.7 |
| BCR4 (Sfru_R3♀ × F1b♂) | 55.7 ± 2.8 |
| Pooled backcross ^d^ | 53.4 ± 2.0 |
|  |  |

^a^ Backcross: Sfru_R3 × F1.

^b^ F2: F1 X F1.

^c^ Number of neonates tested.

^d^ Expected mortality based on inheritance conferred by one or more recessive resistance alleles at one locus, Sfru_R3 homozygous for resistance and SS homozygous for susceptibility: 50% mortality of backcross progeny and 75% mortality of F2.

^e^ Probability from Fisher’s exact test.
