# Supplementary material for: Retrotransposon-mediated disruption of a chitin synthase gene confers insect resistance to Bacillus thuringiensis Vip3Aa toxin
Source: PLoS Biol. 2024 Jul 2;22(7):e3002704. doi: 10.1371/journal.pbio.3002704 (PMC11249258; doi:10.1371/journal.pbio.3002704)
Supplement: S6 Table — (DOCX) [file pbio.3002704.s006.docx]

**S6 Table. Normalized midgut transcript abundance for genes from 8.17 to 8.64 Mb on chromosome 1.**

| Gene ID^a^ | Start (Mb) | End (Mb) | FPKM^b^ | GenBank Annotation^c^ |
| --- | --- | --- | --- | --- |
| **3136** | **8,175,293** | **8,181,479** | **3.43** | **geranylgeranyl transferase type-2 subunit alpha-like** |
| 3137 | 8,182,314 | 8,184,055 | 17.15 | protein abrupt-like |
| **3138** | **8,186,422** | **8,467,817** | **0.28** | **neural-cadherin-like** |
| 3139 | 8,282,422 | 8,284,761 | 0.00 | uncharacterized LOC118273139 |
| 3194 | 8,502,522 | 8,510,565 | 1.02 | formin-like protein 5 |
| **3449** | **8,511,257** | **8,512,953** | **29.36** | **probable 26S proteasome non-ATPase regulatory subunit 3** |
| 3513 | 8,513,304 | 8,514,967 | 0.02 | dynein assembly factor 6, axonemal-like |
| 3659 | 8,519,645 | 8,531,980 | 0.28 | cysteine-rich secretory protein 1-like |
| 3515 | 8,534,307 | 8,549,529 | 0.06 | protein phosphatase PHLPP-like protein |
| 3516 | 8,549,313 | 8,589,130 | 0.00 | LIM/homeobox protein Lhx3-like |
| 3715 | 8,589,783 | 8,595,627 | 0.00 | cilia- and flagella-associated protein 47-like |
| 3406 | 8,598,343 | 8,604,305 | 0.00 | 3,4-dihydroxyphenylacetaldehyde synthase 2-like |
| 3430 | 8,604,300 | 8,605,505 | 1.60 | uncharacterized protein LOC118273407 |
| 3345 | 8,609,025 | 8,610,950 | 9.69 | negative elongation factor E-like |
| 3453 | 8,611,248 | 8,614,886 | 8.87 | cysteine desulfurase, mitochondrial-like |
| 3454 | 8,614,789 | 8,616,366 | 6.54 | ras-related protein Rab-1A-like |
| 3568 | 8,618,802 | 8,623,616 | 0.48 | MPN domain-containing protein CG4751-like |
| **3105** | **8,624,065** | **8,639,856** | **111.97** | **chitin synthase chs-2-like (**)** |
| 3150 | 8,641,476 | 8,668,929 | 1.25 | chitin synthase chs-2 (*) |

^a^ All gene IDs start with LOC11827, only the last four digits are shown above.

^b^ Fragments per kilobase of transcript per million mapped reads.

^c^ Gene ID and GenBank Annotations are from the recent version of the genome of *Spodoptera* *frugiperda*, AGI-APGP_CSIRO_Sfru_2.0 (GCF_023101765.2). (**) = *SfCHS2* chitin synthase 2 (class B) and (*) = *SfCHS1* chitin synthase 1 (class A). The GenBank annotation for 3150 (*SfCHS1*) as chs-2 is not correct.
