# Supplementary material for: Retrotransposon-mediated disruption of a chitin synthase gene confers insect resistance to Bacillus thuringiensis Vip3Aa toxin
Source: PLoS Biol. 2024 Jul 2;22(7):e3002704. doi: 10.1371/journal.pbio.3002704 (PMC11249258; doi:10.1371/journal.pbio.3002704)
Supplement: S7 Table — (DOCX) [file pbio.3002704.s007.docx]

**S7 Table. Responses to Vip3Aa of three SfCHS2-knockout strains and progeny from**

**reciprocal crosses between a knockout strain SfCHS2-KO-A and Sfru_R3.**

| Strain or cross | n^a^ | EC_50_ (95% CI)^b^ | Slope ± SE | RR ^c^ |
| --- | --- | --- | --- | --- |
| SS | 192 | 0.13 (0.11 - 0.16) | 4.0 ± 0.6 | 1.0 |
| SfCHS2-KO-A | 80 | > 1600^d^ | NA^e^ | > 12,000 |
| SfCHS2-KO-B | 96 | > 1600 | NA | > 12,000 |
| SfCHS2-KO-C | 96 | > 1600 | NA | > 12,000 |
| SfCHS2-KO-A♀ × Sfru_R3♂ | 80 | > 1600 | NA | > 12,000 |
| SfCHS2-KO-A♂ × Sfru_R3♀ | 80 | > 1600 | NA | > 12,000 |

^a^ Number of neonates tested.

^b^ Median effective concentration (EC_50_); concentration that caused 50% of neonates to die or fail to advance to the third instar in 7 days and its 95% confidence interval in μg Vip3Aa per cm^2^ diet.

^c^ Resistance ratio; EC_50_ for a strain or progeny from a cross divided by the EC_50_ for SS.

^d^ At the highest concentration tested (1600 μg Vip3Aa per cm^2^ diet), no larvae died or did not develop to third instar (all survived to third instar) for each knockout strain and the progeny from the two reciprocal crosses (n = 10 to 12 per knockout strain or progeny from cross).

^e^ Not available, slope could not be calculated.
