# Supplementary material for: Retrotransposon-mediated disruption of a chitin synthase gene confers insect resistance to Bacillus thuringiensis Vip3Aa toxin
Source: PLoS Biol. 2024 Jul 2;22(7):e3002704. doi: 10.1371/journal.pbio.3002704 (PMC11249258; doi:10.1371/journal.pbio.3002704)
Supplement: S8 Table — (DOCX) [file pbio.3002704.s008.docx]

**S8 Table. Responses to Vip3Aa of *CHS2*-knockout strains of *S. litura* and *M. separata*.**

| Strain | n^a^ | EC_50_ (95% CI)^b^ | Slope ± SE | RR^c^ |
| --- | --- | --- | --- | --- |
| Sl-SS | 96 | 0.016 (0.0030 - 0.086) | 4.1 ± 0.8 | 1.0 |
| SlCHS2-KO | 96 | >1600^d^ | NA^e^ | > 100,000 |
| Ms-SS | 168 | 1.20 (0.97 - 1.48) | 6.6 ± 1.0 | 1.0 |
| MsCHS2-KO | 96 | >1600 | NA | > 1,300 |

^a^ Number of neonates tested.

^b^ Median effective concentration (EC_50_); concentration that caused 50% of neonates to die or fail to advance to the third instar in 7 days and its 95% confidence interval in μg Vip3Aa per cm^2^ diet.

^c^ Resistance ratio; EC_50_ for a strain divided by the EC_50_ for susceptible strain (SS) of the same species.

^d^ At the highest concentration tested (1600 μg Vip3Aa per cm^2^ diet), the percentage of dead larvae plus larvae that did not develop to third instar was 8% for SlCHS2-KO (n = 12) and 0% for MsCHS2-KO (n = 12).

^e^ Not available, slope could not be calculated.
