# Supplementary material for: Retrotransposon-mediated disruption of a chitin synthase gene confers insect resistance to Bacillus thuringiensis Vip3Aa toxin
Source: PLoS Biol. 2024 Jul 2;22(7):e3002704. doi: 10.1371/journal.pbio.3002704 (PMC11249258; doi:10.1371/journal.pbio.3002704)
Supplement: S9 Table — (DOCX) [file pbio.3002704.s009.docx]

| **S9 Table. Individuals of *S. frugiperda* screened for the Yaoer insertion in *SfCHS2*.** | | | | | |
| --- | --- | --- | --- | --- | --- |
|  |  |  |  |  |  |
| **No.** | **ID** | **Country** | **Year** | **Origin** | **Reference** |
| 1 | ArgXXr1 | Argentina | 2018 | lab | Schlum et al. 2021 |
| 2 | ArgXXr2 | Argentina | 2018 | lab | Schlum et al. 2021 |
| 3 | ArgJs1 | Argentina | 2018 | lab | Schlum et al. 2021 |
| 4 | ArgSFu1 | Argentina | 2018 | field | Schlum et al. 2021 |
| 5 | ArgBAu1 | Argentina | 2018 | field | Schlum et al. 2021 |
| 6 | Benin-18 | Benin | 2017 | field | Yainna et al. 2022 |
| 7 | Benin-17 | Benin | 2017 | field | Yainna et al. 2022 |
| 8 | Benin-16 | Benin | 2017 | field | Yainna et al. 2022 |
| 9 | Benin-15 | Benin | 2017 | field | Yainna et al. 2022 |
| 10 | Benin-30 | Benin | 2017 | field | Yainna et al. 2022 |
| 11 | Benin-29 | Benin | 2017 | field | Yainna et al. 2022 |
| 12 | Benin-14 | Benin | 2017 | field | Yainna et al. 2022 |
| 13 | Benin-B2 | Benin | 2017 | field | Yainna et al. 2022 |
| 14 | Benin-B1 | Benin | 2017 | field | Yainna et al. 2022 |
| 15 | Benin-13 | Benin | 2017 | field | Yainna et al. 2022 |
| 16 | Benin-9 | Benin | 2017 | field | Yainna et al. 2022 |
| 17 | Benin-8 | Benin | 2017 | field | Yainna et al. 2022 |
| 18 | Benin-7 | Benin | 2017 | field | Yainna et al. 2022 |
| 19 | Benin-6 | Benin | 2017 | field | Yainna et al. 2022 |
| 20 | Benin-5 | Benin | 2017 | field | Yainna et al. 2022 |
| 21 | Benin-12 | Benin | 2017 | field | Yainna et al. 2022 |
| 22 | Benin-4 | Benin | 2017 | field | Yainna et al. 2022 |
| 23 | Benin-38 | Benin | 2017 | field | Yainna et al. 2022 |
| 24 | Benin-37 | Benin | 2017 | field | Yainna et al. 2022 |
| 25 | Benin-36 | Benin | 2017 | field | Yainna et al. 2022 |
| 26 | Benin-35 | Benin | 2017 | field | Yainna et al. 2022 |
| 27 | Benin-34 | Benin | 2017 | field | Yainna et al. 2022 |
| 28 | Benin-33 | Benin | 2017 | field | Yainna et al. 2022 |
| 29 | Benin-32 | Benin | 2017 | field | Yainna et al. 2022 |
| 30 | Benin-31 | Benin | 2017 | field | Yainna et al. 2022 |
| 31 | Benin-28 | Benin | 2017 | field | Yainna et al. 2022 |
| 32 | Benin-11 | Benin | 2017 | field | Yainna et al. 2022 |
| 33 | Benin-27 | Benin | 2017 | field | Yainna et al. 2022 |
| 34 | Benin-26 | Benin | 2017 | field | Yainna et al. 2022 |
| 35 | Benin-25 | Benin | 2017 | field | Yainna et al. 2022 |
| 36 | Benin-24 | Benin | 2017 | field | Yainna et al. 2022 |
| 37 | Benin-23 | Benin | 2017 | field | Yainna et al. 2022 |
| 38 | Benin-22 | Benin | 2017 | field | Yainna et al. 2022 |
| 39 | Benin-21 | Benin | 2017 | field | Yainna et al. 2022 |
| 40 | Benin-20 | Benin | 2017 | field | Yainna et al. 2022 |
| 41 | Benin-2 | Benin | 2017 | field | Yainna et al. 2022 |
| 42 | Benin-19 | Benin | 2017 | field | Yainna et al. 2022 |
| 43 | Benin-10 | Benin | 2017 | field | Yainna et al. 2022 |
| 44 | Benin-1 | Benin | 2017 | field | Yainna et al. 2022 |
| 45 | BraBAr5 | Brazil | 2018 | lab | Schlum et al. 2021 |
| 46 | BraBAr1 | Brazil | 2018 | lab | Schlum et al. 2021 |
| 47 | BraBAr2 | Brazil | 2018 | lab | Schlum et al. 2021 |
| 48 | BraSPr3 | Brazil | 2018 | lab | Schlum et al. 2021 |
| 49 | BraSPr1 | Brazil | 2018 | lab | Schlum et al. 2021 |
| 50 | BraSPr2 | Brazil | 2018 | lab | Schlum et al. 2021 |
| 51 | BraMGr3 | Brazil | 2018 | lab | Schlum et al. 2021 |
| 52 | BraMGr1 | Brazil | 2018 | lab | Schlum et al. 2021 |
| 53 | BraMGr2 | Brazil | 2018 | lab | Schlum et al. 2021 |
| 54 | BraMGr4 | Brazil | 2018 | lab | Schlum et al. 2021 |
| 55 | BraMGr5 | Brazil | 2018 | lab | Schlum et al. 2021 |
| 56 | BraBAr4 | Brazil | 2018 | lab | Schlum et al. 2021 |
| 57 | BraBAr3 | Brazil | 2018 | lab | Schlum et al. 2021 |
| 58 | JM1 | China | 2020 | field | Zhang et al. 2023 |
| 59 | FYNG173 | China | 2020 | field | Zhang et al. 2023 |
| 60 | FYNG27 | China | 2020 | field | Zhang et al. 2023 |
| 61 | FYNG181 | China | 2020 | field | Zhang et al. 2023 |
| 62 | FYNG167 | China | 2020 | field | Zhang et al. 2023 |
| 63 | GXNN2 | China | 2020 | field | Zhang et al. 2023 |
| 64 | GZQXN3 | China | 2020 | field | Zhang et al. 2023 |
| 65 | GDDD11 | China | 2020 | field | Zhang et al. 2023 |
| 66 | HBWH3 | China | 2020 | field | Zhang et al. 2023 |
| 67 | FYNG28 | China | 2020 | field | Zhang et al. 2023 |
| 68 | GZQXN15 | China | 2020 | field | Zhang et al. 2023 |
| 69 | GZQXN16 | China | 2020 | field | Zhang et al. 2023 |
| 70 | GXNN43 | China | 2020 | field | Zhang et al. 2023 |
| 71 | CQPL1 | China | 2020 | field | Zhang et al. 2023 |
| 72 | GDDD1 | China | 2020 | field | Zhang et al. 2023 |
| 73 | GDDD22 | China | 2020 | field | Zhang et al. 2023 |
| 74 | FYNG182 | China | 2020 | field | Zhang et al. 2023 |
| 75 | GZGY5 | China | 2020 | field | Zhang et al. 2023 |
| 76 | GDDD5 | China | 2020 | field | Zhang et al. 2023 |
| 77 | AHWH4 | China | 2020 | field | Zhang et al. 2023 |
| 78 | JM7 | China | 2020 | field | Zhang et al. 2023 |
| 79 | JM4 | China | 2020 | field | Zhang et al. 2023 |
| 80 | GXNN1 | China | 2020 | field | Zhang et al. 2023 |
| 81 | JM8 | China | 2020 | field | Zhang et al. 2023 |
| 82 | FYNG45 | China | 2020 | field | Zhang et al. 2023 |
| 83 | GZGN7 | China | 2020 | field | Zhang et al. 2023 |
| 84 | GXNN12 | China | 2020 | field | Zhang et al. 2023 |
| 85 | RFAW3 | China | 2020 | field | Zhang et al. 2023 |
| 86 | RFAW2 | China | 2020 | field | Zhang et al. 2023 |
| 87 | GXNN11 | China | 2020 | field | Zhang et al. 2023 |
| 88 | FYNJ16 | China | 2020 | field | Zhang et al. 2023 |
| 89 | GZQXN2 | China | 2020 | field | Zhang et al. 2023 |
| 90 | GZGY20 | China | 2020 | field | Zhang et al. 2023 |
| 91 | JM9 | China | 2020 | field | Zhang et al. 2023 |
| 92 | GXQZ2 | China | 2020 | field | Zhang et al. 2023 |
| 93 | JCX1 | China | 2020 | field | Zhang et al. 2023 |
| 94 | RFAW1 | China | 2020 | field | Zhang et al. 2023 |
| 95 | GXGL2 | China | 2020 | field | Zhang et al. 2023 |
| 96 | CQPL2 | China | 2020 | field | Zhang et al. 2023 |
| 97 | FYNG170 | China | 2020 | field | Zhang et al. 2023 |
| 98 | HBWH2 | China | 2020 | field | Zhang et al. 2023 |
| 99 | GGN1 | China | 2020 | field | Zhang et al. 2023 |
| 100 | FYNJ25 | China | 2020 | field | Zhang et al. 2023 |
| 101 | GDJM2 | China | 2020 | field | Zhang et al. 2023 |
| 102 | HBWX1 | China | 2020 | field | Zhang et al. 2023 |
| 103 | HBWX2 | China | 2020 | field | Zhang et al. 2023 |
| 104 | RFAW4 | China | 2020 | field | Zhang et al. 2023 |
| 105 | ZJWZ61 | China | 2021 | field | Zhang et al. 2023 |
| 106 | YGXL1 | China | 2021 | field | Zhang et al. 2023 |
| 107 | ZJWZ63 | China | 2021 | field | Zhang et al. 2023 |
| 108 | HUBT1 | China | 2021 | field | Zhang et al. 2023 |
| 109 | GDMM7 | China | 2021 | field | Zhang et al. 2023 |
| 110 | YGXG2 | China | 2021 | field | Zhang et al. 2023 |
| 111 | YGXG3 | China | 2021 | field | Zhang et al. 2023 |
| 112 | YHNS4 | China | 2021 | field | Zhang et al. 2023 |
| 113 | GDMM4 | China | 2021 | field | Zhang et al. 2023 |
| 114 | YGXH1 | China | 2021 | field | Zhang et al. 2023 |
| 115 | YHNS3 | China | 2021 | field | Zhang et al. 2023 |
| 116 | YHNS1 | China | 2021 | field | Zhang et al. 2023 |
| 117 | YHNS11 | China | 2021 | field | Zhang et al. 2023 |
| 118 | YHNS13 | China | 2021 | field | Zhang et al. 2023 |
| 119 | YHNS2 | China | 2021 | field | Zhang et al. 2023 |
| 120 | YGXG8 | China | 2021 | field | Zhang et al. 2023 |
| 121 | ZJJH78 | China | 2021 | field | Zhang et al. 2023 |
| 122 | ZJJH64 | China | 2021 | field | Zhang et al. 2023 |
| 123 | GDMM5 | China | 2021 | field | Zhang et al. 2023 |
| 124 | ZJJH63 | China | 2021 | field | Zhang et al. 2023 |
| 125 | YHNH2 | China | 2021 | field | Zhang et al. 2023 |
| 126 | YHNH1 | China | 2021 | field | Zhang et al. 2023 |
| 127 | ZJWZ2 | China | 2021 | field | Zhang et al. 2023 |
| 128 | HUBT2 | China | 2021 | field | Zhang et al. 2023 |
| 129 | GDMM12 | China | 2021 | field | Zhang et al. 2023 |
| 130 | YGXL9 | China | 2021 | field | Zhang et al. 2023 |
| 131 | YGXG1 | China | 2021 | field | Zhang et al. 2023 |
| 132 | YGXH4 | China | 2021 | field | Zhang et al. 2023 |
| 133 | YGXL2 | China | 2021 | field | Zhang et al. 2023 |
| 134 | YHNH8 | China | 2021 | field | Zhang et al. 2023 |
| 135 | YHNH9 | China | 2021 | field | Zhang et al. 2023 |
| 136 | GDMM13 | China | 2021 | field | Zhang et al. 2023 |
| 137 | SLS2 | China | 2019 | field | Zhang et al. 2020 |
| 138 | SLS1 | China | 2019 | field | Zhang et al. 2020 |
| 139 | JHM1 | China | 2019 | field | Zhang et al. 2020 |
| 140 | HSY2 | China | 2019 | field | Zhang et al. 2020 |
| 141 | AWJ2 | China | 2019 | field | Zhang et al. 2020 |
| 142 | GLZ1 | China | 2019 | field | Zhang et al. 2020 |
| 143 | XLZ1 | China | 2019 | field | Zhang et al. 2020 |
| 144 | GGY1 | China | 2019 | field | Zhang et al. 2020 |
| 145 | JNC2 | China | 2019 | field | Zhang et al. 2020 |
| 146 | JNC1 | China | 2019 | field | Zhang et al. 2020 |
| 147 | GLZ3 | China | 2019 | field | Zhang et al. 2020 |
| 148 | SFX1 | China | 2019 | field | Zhang et al. 2020 |
| 149 | CXS2 | China | 2019 | field | Zhang et al. 2020 |
| 150 | CXS1 | China | 2019 | field | Zhang et al. 2020 |
| 151 | AWJ1 | China | 2019 | field | Zhang et al. 2020 |
| 152 | YZ2 | China | 2019 | field | Zhang et al. 2020 |
| 153 | HYC1 | China | 2019 | field | Zhang et al. 2020 |
| 154 | YKM1 | China | 2019 | field | Zhang et al. 2020 |
| 155 | YZ1 | China | 2019 | field | Zhang et al. 2020 |
| 156 | SHZ1 | China | 2019 | field | Zhang et al. 2020 |
| 157 | ZLQ1 | China | 2019 | field | Zhang et al. 2020 |
| 158 | HYC2 | China | 2019 | field | Zhang et al. 2020 |
| 159 | GWY1 | China | 2019 | field | Zhang et al. 2020 |
| 160 | GGY2 | China | 2019 | field | Zhang et al. 2020 |
| 161 | HSY1 | China | 2019 | field | Zhang et al. 2020 |
| 162 | SXX1 | China | 2019 | field | Zhang et al. 2020 |
| 163 | GWY2 | China | 2019 | field | Zhang et al. 2020 |
| 164 | YKM2 | China | 2019 | field | Zhang et al. 2020 |
| 165 | HAN201 | China | 2022 | field | this study |
| 166 | HAN202 | China | 2022 | field | this study |
| 167 | HAN208 | China | 2022 | field | this study |
| 168 | HBTS3 | China | 2022 | field | this study |
| 169 | HBTS5 | China | 2022 | field | this study |
| 170 | HBTS6 | China | 2022 | field | this study |
| 171 | HBTS7 | China | 2022 | field | this study |
| 172 | HBTS1 | China | 2022 | field | this study |
| 173 | HBTS2 | China | 2022 | field | this study |
| 174 | HBTS4 | China | 2022 | field | this study |
| 175 | ZJSX1 | China | 2022 | field | this study |
| 176 | ZJSX3 | China | 2022 | field | this study |
| 177 | ZJSX4 | China | 2022 | field | this study |
| 178 | ZJSX6 | China | 2022 | field | this study |
| 179 | ZJSX7 | China | 2022 | field | this study |
| 180 | GXHC201 | China | 2022 | field | this study |
| 181 | GXHC202 | China | 2022 | field | this study |
| 182 | GXHC203 | China | 2022 | field | this study |
| 183 | GXHC204 | China | 2022 | field | this study |
| 184 | GXHC205 | China | 2022 | field | this study |
| 185 | FJYX1 | China | 2020 | field | this study |
| 186 | FJYX2 | China | 2020 | field | this study |
| 187 | FJYX6 | China | 2020 | field | this study |
| 188 | FJYX7 | China | 2020 | field | this study |
| 189 | FJYX16 | China | 2020 | field | this study |
| 190 | FJYA19 | China | 2020 | field | this study |
| 191 | FJYA20 | China | 2020 | field | this study |
| 192 | FJYA21 | China | 2020 | field | this study |
| 193 | FJYA22 | China | 2020 | field | this study |
| 194 | FJYA23 | China | 2020 | field | this study |
| 195 | FJYA3 | China | 2020 | field | this study |
| 196 | GXNN10 | China | 2020 | field | this study |
| 197 | GXNN13 | China | 2020 | field | this study |
| 198 | GXNN14 | China | 2020 | field | this study |
| 199 | GDYJ3 | China | 2020 | field | this study |
| 200 | GDYJ4 | China | 2020 | field | this study |
| 201 | GDYJ5 | China | 2020 | field | this study |
| 202 | GDYJ6 | China | 2020 | field | this study |
| 203 | GDYJ7 | China | 2020 | field | this study |
| 204 | GDJM9 | China | 2020 | field | this study |
| 205 | GDJM10 | China | 2020 | field | this study |
| 206 | GDJM11 | China | 2020 | field | this study |
| 207 | GDJM12 | China | 2020 | field | this study |
| **208** | **GDJM14** | **China** | **2020** | **field** | **this study** |
| 209 | GDJM109 | China | 2022 | field | this study |
| 210 | GDJM114 | China | 2022 | field | this study |
| 211 | GDJM115 | China | 2022 | field | this study |
| 212 | GDJM116 | China | 2022 | field | this study |
| 213 | GDJM117 | China | 2022 | field | this study |
| 214 | HBWX3 | China | 2020 | field | this study |
| 215 | ZLS2 | China | 2019 | field | Zhang et al. 2020 |
| 216 | Ethiopia-504 | Ethiopia | 2019 | field | Gui et al. 2022 |
| 217 | Ethiopia-505 | Ethiopia | 2019 | field | Gui et al. 2022 |
| 218 | Ethiopia-577 | Ethiopia | 2019 | field | Gui et al. 2022 |
| 219 | Ethiopia-578 | Ethiopia | 2019 | field | Gui et al. 2022 |
| 220 | Ethiopia-579 | Ethiopia | 2019 | field | Gui et al. 2022 |
| 221 | Ethiopia-501 | Ethiopia | 2019 | field | Gui et al. 2022 |
| 222 | Ethiopia-506 | Ethiopia | 2019 | field | Gui et al. 2022 |
| 223 | Ethiopia-503 | Ethiopia | 2019 | field | Gui et al. 2022 |
| 224 | Ethiopia-576 | Ethiopia | 2019 | field | Gui et al. 2022 |
| 225 | Ethiopia-502 | Ethiopia | 2019 | field | Gui et al. 2022 |
| 226 | Gua2-3 | France | 2013 | field | Yainna et al. 2022 |
| 227 | Gua2-2 | France | 2013 | field | Yainna et al. 2022 |
| 228 | Gua1-2 | France | 2013 | field | Yainna et al. 2022 |
| 229 | Gua1-1 | France | 2013 | field | Yainna et al. 2022 |
| 230 | GuiFG4 | French Guiana | 1992 | / | Yainna et al. 2022 |
| 231 | GuiFG3 | French Guiana | 1992 | / | Yainna et al. 2022 |
| 232 | GuiFG2 | French Guiana | 1992 | / | Yainna et al. 2022 |
| 233 | AGH-5 | Ghana | 2017 | field | Zhang et al. 2023 |
| 234 | AGH-2 | Ghana | 2017 | field | Zhang et al. 2023 |
| 235 | AGH-1 | Ghana | 2017 | field | Zhang et al. 2023 |
| 236 | AGH-6 | Ghana | 2017 | field | Zhang et al. 2023 |
| 237 | AGH-4 | Ghana | 2017 | field | Zhang et al. 2023 |
| 238 | AGH-8 | Ghana | 2017 | field | Zhang et al. 2023 |
| 239 | AGH-9 | Ghana | 2017 | field | Zhang et al. 2023 |
| 240 | AGH-3 | Ghana | 2017 | field | Zhang et al. 2023 |
| 241 | AGH-7 | Ghana | 2017 | field | Zhang et al. 2023 |
| 242 | AGH-10 | Ghana | 2017 | field | Zhang et al. 2023 |
| 243 | Ghaucc1 | Ghana | 2017 | field | this study |
| 244 | Ghaucc2 | Ghana | 2017 | field | this study |
| 245 | Ghaucc5 | Ghana | 2017 | field | this study |
| 246 | Ghaucc7 | Ghana | 2017 | field | this study |
| 247 | GhaTA1 | Ghana | 2017 | field | this study |
| 248 | GhaTA3 | Ghana | 2017 | field | this study |
| 249 | GhaTA7 | Ghana | 2017 | field | this study |
| 250 | GhaTA10 | Ghana | 2017 | field | this study |
| 251 | Gha3 | Ghana | 2017 | field | this study |
| 252 | Gha4 | Ghana | 2017 | field | this study |
| 253 | Gha5 | Ghana | 2017 | field | this study |
| 254 | Gha6 | Ghana | 2017 | field | this study |
| 255 | Gha7 | Ghana | 2017 | field | this study |
| 256 | Gha8 | Ghana | 2017 | field | this study |
| 257 | ASW2 | Guadeloupe | 2000 | lab | Gouin et al. 2017 |
| 258 | ASW6 | Guadeloupe | 2000 | lab | Gouin et al. 2017 |
| 259 | ASW7 | Guadeloupe | 2000 | lab | Gouin et al. 2017 |
| 260 | Ind-S3 | India | 2018 | field | Yainna et al. 2022 |
| 261 | Ind-M1a | India | 2018 | field | Yainna et al. 2022 |
| 262 | Ind-F1a | India | 2018 | field | Yainna et al. 2022 |
| 263 | Ind-B9 | India | 2018 | field | Yainna et al. 2022 |
| 264 | Ind-B8 | India | 2018 | field | Yainna et al. 2022 |
| 265 | Ind-B7 | India | 2018 | field | Yainna et al. 2022 |
| 266 | Ind-B6 | India | 2018 | field | Yainna et al. 2022 |
| 267 | Ind-B5 | India | 2018 | field | Yainna et al. 2022 |
| 268 | Ind-B4 | India | 2018 | field | Yainna et al. 2022 |
| 269 | Ind-B3 | India | 2018 | field | Yainna et al. 2022 |
| 270 | Ind-B2 | India | 2018 | field | Yainna et al. 2022 |
| 271 | Ind-B12 | India | 2018 | field | Yainna et al. 2022 |
| 272 | Ind-B10 | India | 2018 | field | Yainna et al. 2022 |
| 273 | Ind-B1 | India | 2018 | field | Yainna et al. 2022 |
| 274 | KenXXu3 | Kenya | 2019 | lab | Schlum et al. 2021 |
| 275 | KenXXu1 | Kenya | 2019 | lab | Schlum et al. 2021 |
| 276 | KenXXu2 | Kenya | 2019 | lab | Schlum et al. 2021 |
| 277 | Ken26 | Kenya | 2017 | field | this study |
| 278 | Ken27 | Kenya | 2017 | field | this study |
| 279 | Ken30 | Kenya | 2017 | field | this study |
| 280 | Kenya-507 | Kenya | 2018 | field | Gui et al. 2022 |
| 281 | Kenya-516 | Kenya | 2018 | field | Gui et al. 2022 |
| 282 | Kenya-509 | Kenya | 2018 | field | Gui et al. 2022 |
| 283 | Kenya-510 | Kenya | 2018 | field | Gui et al. 2022 |
| 284 | Kenya-511 | Kenya | 2018 | field | Gui et al. 2022 |
| 285 | Kenya-512 | Kenya | 2018 | field | Gui et al. 2022 |
| 286 | Kenya-513 | Kenya | 2018 | field | Gui et al. 2022 |
| 287 | Kenya-514 | Kenya | 2018 | field | Gui et al. 2022 |
| 288 | Kenya-515 | Kenya | 2018 | field | Gui et al. 2022 |
| 289 | AMA-9 | Malawi | 2017 | field | Zhang et al. 2023 |
| 290 | AMA-4 | Malawi | 2017 | field | Zhang et al. 2023 |
| 291 | AMA-5 | Malawi | 2017 | field | Zhang et al. 2023 |
| 292 | AMA-7 | Malawi | 2017 | field | Zhang et al. 2023 |
| 293 | AMA-1 | Malawi | 2017 | field | Zhang et al. 2023 |
| 294 | AMA-8 | Malawi | 2017 | field | Zhang et al. 2023 |
| 295 | AMA-3 | Malawi | 2017 | field | Zhang et al. 2023 |
| 296 | AMA-2 | Malawi | 2017 | field | Zhang et al. 2023 |
| 297 | AMA-10 | Malawi | 2017 | field | Zhang et al. 2023 |
| 298 | AMA-6 | Malawi | 2017 | field | Zhang et al. 2023 |
| 299 | MalT7 | Malawi | 2017 | field | this study |
| 300 | MalT8 | Malawi | 2017 | field | this study |
| 301 | MalT9 | Malawi | 2017 | field | this study |
| 302 | MalT16 | Malawi | 2017 | field | this study |
| 303 | MalT18 | Malawi | 2017 | field | this study |
| 304 | MalT19 | Malawi | 2017 | field | this study |
| 305 | MAL-1 | Malaysia | 2020 | field | Zhang et al. 2023 |
| 306 | MAL-4 | Malaysia | 2020 | field | Zhang et al. 2023 |
| 307 | MAL-5 | Malaysia | 2020 | field | Zhang et al. 2023 |
| 308 | MAL-3 | Malaysia | 2020 | field | Zhang et al. 2023 |
| 309 | MAL-2 | Malaysia | 2020 | field | Zhang et al. 2023 |
| 310 | Mex9 | Mexico | 2009 | field | Yainna et al. 2022 |
| 311 | Mex8 | Mexico | 2009 | field | Yainna et al. 2022 |
| 312 | Mex6 | Mexico | 2009 | field | Yainna et al. 2022 |
| 313 | Mex38 | Mexico | 2009 | field | Yainna et al. 2022 |
| 314 | Mex35 | Mexico | 2009 | field | Yainna et al. 2022 |
| 315 | Mex33 | Mexico | 2009 | field | Yainna et al. 2022 |
| 316 | Mex31 | Mexico | 2009 | field | Yainna et al. 2022 |
| 317 | Mex28 | Mexico | 2009 | field | Yainna et al. 2022 |
| 318 | Mex27 | Mexico | 2009 | field | Yainna et al. 2022 |
| 319 | Mex25 | Mexico | 2009 | field | Yainna et al. 2022 |
| 320 | Mex23 | Mexico | 2009 | field | Yainna et al. 2022 |
| 321 | Mex21 | Mexico | 2009 | field | Yainna et al. 2022 |
| 322 | Mex18 | Mexico | 2009 | field | Yainna et al. 2022 |
| 323 | Mex16 | Mexico | 2009 | field | Yainna et al. 2022 |
| 324 | Mex15 | Mexico | 2009 | field | Yainna et al. 2022 |
| 325 | Mex13 | Mexico | 2009 | field | Yainna et al. 2022 |
| 326 | Mex48 | Mexico | 2009 | field | Yainna et al. 2022 |
| 327 | Mex47 | Mexico | 2009 | field | Yainna et al. 2022 |
| 328 | Mex46 | Mexico | 2009 | field | Yainna et al. 2022 |
| 329 | Mex45 | Mexico | 2009 | field | Yainna et al. 2022 |
| 330 | Mex44 | Mexico | 2009 | field | Yainna et al. 2022 |
| 331 | Mex43 | Mexico | 2009 | field | Yainna et al. 2022 |
| 332 | Mex42 | Mexico | 2009 | field | Yainna et al. 2022 |
| 333 | Mex41 | Mexico | 2009 | field | Yainna et al. 2022 |
| 334 | Mex40 | Mexico | 2009 | field | Yainna et al. 2022 |
| 335 | Mex39 | Mexico | 2009 | field | Yainna et al. 2022 |
| 336 | PueSIu1 | Puerto Rico | 2019 | field | Schlum et al. 2021 |
| 337 | PueLAu1 | Puerto Rico | 2019 | field | Schlum et al. 2021 |
| 338 | PueGUr1 | Puerto Rico | 2019 | field | Schlum et al. 2021 |
| 339 | PR32 | Puerto Rico | 2009 | field | Gimenez et al. 2021 |
| 340 | PR27 | Puerto Rico | 2009 | field | Gimenez et al. 2021 |
| 341 | PR14 | Puerto Rico | 2009 | field | Gimenez et al. 2021 |
| 342 | PR35 | Puerto Rico | 2009 | field | Gimenez et al. 2021 |
| 343 | PR30 | Puerto Rico | 2009 | field | Gimenez et al. 2021 |
| 344 | PR15 | Puerto Rico | 2009 | field | Gimenez et al. 2021 |
| 345 | PR18 | Puerto Rico | 2009 | field | Gimenez et al. 2021 |
| 346 | PR5 | Puerto Rico | 2009 | field | Gimenez et al. 2021 |
| 347 | PR33 | Puerto Rico | 2009 | field | Gimenez et al. 2021 |
| 348 | PR31 | Puerto Rico | 2009 | field | Gimenez et al. 2021 |
| 349 | PR29 | Puerto Rico | 2009 | field | Gimenez et al. 2021 |
| 350 | PR16 | Puerto Rico | 2009 | field | Gimenez et al. 2021 |
| 351 | PR12 | Puerto Rico | 2009 | field | Gimenez et al. 2021 |
| 352 | PR1 | Puerto Rico | 2009 | field | Gimenez et al. 2021 |
| 353 | PR19 | Puerto Rico | 2009 | field | Gimenez et al. 2021 |
| 354 | RWA-213 | Rwanda | 2017 | field | Zhang et al. 2023 |
| 355 | RWA-201 | Rwanda | 2017 | field | Zhang et al. 2023 |
| 356 | RWA-221 | Rwanda | 2017 | field | Zhang et al. 2023 |
| 357 | RWA-222 | Rwanda | 2017 | field | Zhang et al. 2023 |
| 358 | RWA-202 | Rwanda | 2017 | field | Zhang et al. 2023 |
| 359 | RWA-208 | Rwanda | 2017 | field | Zhang et al. 2023 |
| 360 | Rwa72 | Rwanda | 2017 | field | this study |
| 361 | Rwa119 | Rwanda | 2017 | field | this study |
| 362 | Rwa214 | Rwanda | 2017 | field | this study |
| 363 | Rwa71A | Rwanda | 2017 | field | this study |
| 364 | Rwa108B | Rwanda | 2017 | field | this study |
| 365 | Rwa109A | Rwanda | 2017 | field | this study |
| 366 | Rwa1B | Rwanda | 2017 | field | this study |
| 367 | Rwa9B | Rwanda | 2017 | field | this study |
| 368 | Rwa29B | Rwanda | 2017 | field | this study |
| 369 | Rwa51B | Rwanda | 2017 | field | this study |
| 370 | Rwa9C | Rwanda | 2017 | field | this study |
| 371 | Rwa29C | Rwanda | 2017 | field | this study |
| 372 | Rwa127C | Rwanda | 2017 | field | this study |
| 373 | Rwa52D | Rwanda | 2017 | field | this study |
| 374 | Rwa54D | Rwanda | 2017 | field | this study |
| 375 | Rwa22E | Rwanda | 2017 | field | this study |
| 376 | Rwa54E | Rwanda | 2017 | field | this study |
| 377 | Rwa94H | Rwanda | 2017 | field | this study |
| 378 | SouthA-524 | South Africa | 2019 | field | Gui et al. 2022 |
| 379 | SouthA-522 | South Africa | 2019 | field | Gui et al. 2022 |
| 380 | SouthA-533 | South Africa | 2019 | field | Gui et al. 2022 |
| 381 | SouthA-523 | South Africa | 2019 | field | Gui et al. 2022 |
| 382 | ASU-8 | Sudan | 2017 | field | Zhang et al. 2023 |
| 383 | ASU-5 | Sudan | 2017 | field | Zhang et al. 2023 |
| 384 | ASU12 | Sudan | 2017 | field | Zhang et al. 2023 |
| 385 | Sudc2H | Sudan | 2017 | field | this study |
| 386 | Sudc5H | Sudan | 2017 | field | this study |
| 387 | Sudc6H | Sudan | 2017 | field | this study |
| 388 | Sudc7H | Sudan | 2017 | field | this study |
| 389 | Sudc10H | Sudan | 2017 | field | this study |
| 390 | MS-R6 | USA | 2009 | field | Gimenez et al. 2021 |
| 391 | MS-C7 | USA | 2009 | field | Gimenez et al. 2021 |
| 392 | MS-C4 | USA | 2009 | field | Gimenez et al. 2021 |
| 393 | MS-C2 | USA | 2009 | field | Gimenez et al. 2021 |
| 394 | MS-C3 | USA | 2009 | field | Gimenez et al. 2021 |
| 395 | MS-C5 | USA | 2009 | field | Gimenez et al. 2021 |
| 396 | MS-C6 | USA | 2009 | field | Gimenez et al. 2021 |
| 397 | MS-C1 | USA | 2009 | field | Gimenez et al. 2021 |
| 398 | MS-C8 | USA | 2009 | field | Gimenez et al. 2021 |
| 399 | MS-C9 | USA | 2009 | field | Gimenez et al. 2021 |
| 400 | MS-R9 | USA | 2009 | field | Gimenez et al. 2021 |
| 401 | MS-R1 | USA | 2009 | field | Gimenez et al. 2021 |
| 402 | MS-R4 | USA | 2009 | field | Gimenez et al. 2021 |
| 403 | MS-R5 | USA | 2009 | field | Gimenez et al. 2021 |
| 404 | MS-R2 | USA | 2009 | field | Gimenez et al. 2021 |
| 405 | MS-R3 | USA | 2009 | field | Gimenez et al. 2021 |
| 406 | MS-R7 | USA | 2009 | field | Gimenez et al. 2021 |
| 407 | MS-R8 | USA | 2009 | field | Gimenez et al. 2021 |
| 408 | AXE3 | USA | 2010 | lab | Gouin et al. 2017 |
| 409 | AXE5 | USA | 2010 | lab | Gouin et al. 2017 |
| 410 | AXE6 | USA | 2010 | lab | Gouin et al. 2017 |
| 411 | USAFLu2 | USA | 2016 | field | Schlum et al. 2021 |
| 412 | USAC-558 | USA | 2016 | lab | Gui et al. 2022 |
| 413 | USAC-555 | USA | 2016 | lab | Gui et al. 2022 |
| 414 | USAC-560 | USA | 2016 | lab | Gui et al. 2022 |
| 415 | USAC-562 | USA | 2016 | lab | Gui et al. 2022 |
| 416 | USAC-563 | USA | 2016 | lab | Gui et al. 2022 |
| 417 | USAC-561 | USA | 2016 | lab | Gui et al. 2022 |
| 418 | USAC-557 | USA | 2016 | lab | Gui et al. 2022 |
| 419 | USAC-556 | USA | 2016 | lab | Gui et al. 2022 |
| 420 | USANCr1 | USA | 2016 | lab | Schlum et al. 2021 |
| 421 | USAFLr2 | USA | 2016 | lab | Schlum et al. 2021 |
| 422 | USAFLr1 | USA | 2016 | lab | Schlum et al. 2021 |
| 423 | USAMSs2 | USA | 2016 | lab | Schlum et al. 2021 |
| 424 | USAFLu9 | USA | 2016 | field | Schlum et al. 2021 |
| 425 | USAFLu12 | USA | 2016 | field | Schlum et al. 2021 |
| 426 | USAFLu8 | USA | 2016 | field | Schlum et al. 2021 |
| 427 | USAFLu10 | USA | 2016 | field | Schlum et al. 2021 |
| 428 | USAFLu11 | USA | 2016 | field | Schlum et al. 2021 |
| 429 | USATXu1 | USA | 2017 | field | Schlum et al. 2021 |
| 430 | USATXu2 | USA | 2017 | field | Schlum et al. 2021 |
| 431 | USATXu3 | USA | 2017 | field | Schlum et al. 2021 |
| 432 | USATXu5 | USA | 2017 | field | Schlum et al. 2021 |
| 433 | USATXu4 | USA | 2017 | field | Schlum et al. 2021 |
| 434 | USAMAu1 | USA | 2017 | field | Schlum et al. 2021 |
| 435 | USAMAu2 | USA | 2017 | field | Schlum et al. 2021 |
| 436 | USAB-548 | USA | 2017 | lab | Gui et al. 2022 |
| 437 | USAB-547 | USA | 2017 | lab | Gui et al. 2022 |
| 438 | USAB-549 | USA | 2017 | lab | Gui et al. 2022 |
| 439 | USAB-545 | USA | 2017 | lab | Gui et al. 2022 |
| 440 | USAB-552 | USA | 2017 | lab | Gui et al. 2022 |
| 441 | USAB-550 | USA | 2017 | lab | Gui et al. 2022 |
| 442 | USAB-554 | USA | 2017 | lab | Gui et al. 2022 |
| 443 | USAB-546 | USA | 2017 | lab | Gui et al. 2022 |
| 444 | USAB-553 | USA | 2017 | lab | Gui et al. 2022 |
| 445 | USAB-551 | USA | 2017 | lab | Gui et al. 2022 |
| 446 | USATXu6 | USA | 2017 | field | Schlum et al. 2021 |
| 447 | USAMNu1 | USA | 2017 | field | Schlum et al. 2021 |
| 448 | USAMNu2 | USA | 2017 | field | Schlum et al. 2021 |
| 449 | USAFLu3 | USA | 2017 | field | Schlum et al. 2021 |
| 450 | USAFLu6 | USA | 2017 | field | Schlum et al. 2021 |
| 451 | USASCu1 | USA | 2017 | field | Schlum et al. 2021 |
| 452 | USASCu2 | USA | 2017 | field | Schlum et al. 2021 |
| 453 | USAFLu1 | USA | 2017 | field | Schlum et al. 2021 |
| 454 | USAFLu5 | USA | 2017 | field | Schlum et al. 2021 |
| 455 | USAFLu4 | USA | 2017 | field | Schlum et al. 2021 |
| 456 | USAFLu7 | USA | 2017 | field | Schlum et al. 2021 |
| 457 | USATNu1 | USA | 2018 | field | Schlum et al. 2021 |
| 458 | USATNu2 | USA | 2018 | field | Schlum et al. 2021 |
| 459 | USAA-543 | USA | 2019 | lab | Gui et al. 2022 |
| 460 | USAA-539 | USA | 2019 | lab | Gui et al. 2022 |
| 461 | USAA-544 | USA | 2019 | lab | Gui et al. 2022 |
| 462 | USAA-535 | USA | 2019 | lab | Gui et al. 2022 |
| 463 | USAA-536 | USA | 2019 | lab | Gui et al. 2022 |
| 464 | USAA-538 | USA | 2019 | lab | Gui et al. 2022 |
| 465 | USAA-542 | USA | 2019 | lab | Gui et al. 2022 |
| 466 | USAA-537 | USA | 2019 | lab | Gui et al. 2022 |
| 467 | USAA-540 | USA | 2019 | lab | Gui et al. 2022 |
| 468 | USAA-541 | USA | 2019 | lab | Gui et al. 2022 |
| 469 | USAMSs1 | USA | / | lab | Schlum et al. 2021 |
| 470 | USA-LaW6 | USA | 2021 | lab | this study |
| 471 | USA-LaW8 | USA | 2021 | lab | this study |
| 472 | USA-Ms6 | USA | 2021 | lab | this study |
| 473 | USA-Ms8 | USA | 2021 | lab | this study |
| 474 | USA-LaW1 | USA | 2021 | lab | this study |
| 475 | USA-LaW2 | USA | 2021 | lab | this study |
| 476 | USA-LaW3 | USA | 2021 | lab | this study |
| 477 | USA-LaW4 | USA | 2021 | lab | this study |
| 478 | USA-LaW5 | USA | 2021 | lab | this study |
| 479 | USA-LaW7 | USA | 2021 | lab | this study |
| 480 | USA-LaW9 | USA | 2021 | lab | this study |
| 481 | USA-LaW10 | USA | 2021 | lab | this study |
| 482 | USA-LaW11 | USA | 2021 | lab | this study |
| 483 | USA-LaW12 | USA | 2021 | lab | this study |
| 484 | USA-LaW13 | USA | 2021 | lab | this study |
| 485 | USA-LaW14 | USA | 2021 | lab | this study |
| 486 | USA-LaW20 | USA | 2021 | lab | this study |
| 487 | USA-Ms1 | USA | / | lab | this study |
| 488 | USA-Ms2 | USA | / | lab | this study |
| 489 | USA-Ms3 | USA | / | lab | this study |
| 490 | USA-Ms5 | USA | / | lab | this study |
| 491 | USA-Ms7 | USA | / | lab | this study |
| 492 | USA-Ms9 | USA | / | lab | this study |
| 493 | USA-Ms10 | USA | / | lab | this study |
| 494 | USA-Ms11 | USA | / | lab | this study |
| 495 | USA-Ms12 | USA | / | lab | this study |
| 496 | USA-Ms18 | USA | / | lab | this study |
| 497 | USAFGJ3 | USA | 2015 | field | Fiteni et al. 2022 |
| 498 | USAFGJ5 | USA | 2015 | field | Fiteni et al. 2022 |
| 499 | USAFGJ6 | USA | 2015 | field | Fiteni et al. 2022 |
| 500 | USAFGJ7 | USA | 2015 | field | Fiteni et al. 2022 |
| 501 | USAFGJ8 | USA | 2015 | field | Fiteni et al. 2022 |
| 502 | USAFGJ9 | USA | 2015 | field | Fiteni et al. 2022 |
| 503 | USAFCC1 | USA | 2015 | field | Fiteni et al. 2022 |
| 504 | USAFCC5 | USA | 2015 | field | Fiteni et al. 2022 |
| 505 | USAFCC6 | USA | 2015 | field | Fiteni et al. 2022 |
| 506 | USAFCC7 | USA | 2015 | field | Fiteni et al. 2022 |
| 507 | USAFCC8 | USA | 2015 | field | Fiteni et al. 2022 |
| 508 | USAFCC2 | USA | 2015 | field | Fiteni et al. 2022 |
| 509 | USAFL16 | USA | 2015 | field | Fiteni et al. 2022 |
| 510 | USAFL17 | USA | 2015 | field | Fiteni et al. 2022 |
| 511 | USAFL18 | USA | 2015 | field | Fiteni et al. 2022 |
| 512 | USAFL19 | USA | 2015 | field | Fiteni et al. 2022 |
| 513 | USAFL20 | USA | 2015 | field | Fiteni et al. 2022 |
| 514 | USAFGJ4 | USA | 2015 | field | Fiteni et al. 2022 |
| 515 | USAFGJ10 | USA | 2015 | field | Fiteni et al. 2022 |
| 516 | USAFGJ11 | USA | 2015 | field | Fiteni et al. 2022 |
| 517 | USAFGJ12 | USA | 2015 | field | Fiteni et al. 2022 |
| 518 | USAFGJ2 | USA | 2015 | field | Fiteni et al. 2022 |
| 519 | USAFCC3 | USA | 2015 | field | Fiteni et al. 2022 |
| 520 | USAFCC4 | USA | 2015 | field | Fiteni et al. 2022 |
| 521 | AFR2 | Zambia | 2017 | lab | Zhang et al. 2023 |
| 522 | AFR7 | Zambia | 2017 | lab | Zhang et al. 2023 |
| 523 | AFR10 | Zambia | 2017 | lab | Zhang et al. 2023 |
| 524 | AFR6 | Zambia | 2017 | lab | Zhang et al. 2023 |
| 525 | AFR9 | Zambia | 2017 | lab | Zhang et al. 2023 |
| 526 | AFR5 | Zambia | 2017 | lab | Zhang et al. 2023 |
| 527 | AFR1 | Zambia | 2017 | lab | Zhang et al. 2023 |
| 528 | AFR4 | Zambia | 2017 | lab | Zhang et al. 2023 |
| 529 | AFR3 | Zambia | 2017 | lab | Zhang et al. 2023 |
| 530 | AFR8 | Zambia | 2017 | lab | Zhang et al. 2023 |
| 531 | ALA-6 | Zambia | 2017 | field | Zhang et al. 2023 |
| 532 | ZamI2 | Zambia | 2017 | field | this study |
| 533 | ZamI4 | Zambia | 2017 | field | this study |
| 534 | ZamI5 | Zambia | 2017 | field | this study |
| 535 | ZamI7 | Zambia | 2017 | field | this study |
| 536 | ZamG1 | Zambia | 2017 | field | this study |
| 537 | ZamG3 | Zambia | 2017 | field | this study |
| 538 | ZamG6 | Zambia | 2017 | field | this study |
| 539 | ZamG10 | Zambia | 2017 | field | this study |
| 540 | ZamH6 | Zambia | 2017 | field | this study |
|  |  |  |  |  |  |
| **References**   1. Fiteni E, Durand K, Gimenez S, Meagher RL, Legeai F, Kergoat GJ, et al. Host-plant adaptation as a driver of incipient speciation in the fall armyworm (*Spodoptera frugiperda*). Bmc Ecol Evol. 2022;22(1). doi: ARTN 13310.1186/s12862-022-02090-x. WOS:000881986400001. 2. Gimenez S, Abdelgaffar H, Goff GL, Hilliou F, Blanco CA, Hänniger S, et al. Adaptation by copy number variation increases insecticide resistance in the fall armyworm. Commun Biol. 2020;3(1). doi: ARTN 66410.1038/s42003-020-01382-6. WOS:000593980600002. 3. Gouin A, Bretaudeau A, Nam K, Gimenez S, Aury JM, Duvic B, et al. Two genomes of highly polyphagous lepidopteran pests (*Spodoptera frugiperd*a, Noctuidae) with different host-plant ranges. Sci Rep. 2017;7. doi: ARTN 1181610.1038/s41598-017-10461-4. WOS:000411647900001. 4. Gui FR, Lan TM, Zhao Y, Guo W, Dong Y, Fang DM, et al. Genomic and transcriptomic analysis unveils population evolution and development of pesticide resistance in fall armyworm. Protein Cell. 2022;13(7):513-31. doi: 10.1007/s13238-020-00795-7. WOS:000582768400001. 5. Schlum KA, Lamour K, de Bortoli CP, Banerjee R, Meagher R, Pereira E, et al. Whole genome comparisons reveal panmixia among fall armyworm (*Spodoptera frugiperda*) from diverse locations. BMC Genomics. 2021;22(1). doi: ARTN 17910.1186/s12864-021-07492-7. WOS:000628995700003. 6. Yainna S, Tay WT, Durand K, Fiteni E, Hilliou F, Legeai F, et al. The evolutionary process of invasion in the fall armyworm (*Spodoptera frugiperda*). Sci Rep. 2022;12(1). doi: ARTN 21063 10.1038/s41598-022-25529-z. WOS:000934498000021. 7. Zhang L, Li ZY, Peng Y, Liang XY, Wilson K, Chipabika G, et al. Global genomic signature reveals the evolution of fall armyworm in the Eastern hemisphere. Mol Ecol. 2023;32(20):5463-78. doi: 10.1111/mec.17117. WOS:001080612500001. 8. Zhang L, Liu B, Zheng WG, Liu CH, Zhang DN, Zhao SY, et al. Genetic structure and insecticide resistance characteristics of fall armyworm populations invading China. Mol Ecol Resour. 2020;20(6):1682-96. doi: 10.1111/1755-0998.13219. WOS:000550472500001. | | | | | |
