# Supplementary material for: Retrotransposon-mediated disruption of a chitin synthase gene confers insect resistance to Bacillus thuringiensis Vip3Aa toxin
Source: PLoS Biol. 2024 Jul 2;22(7):e3002704. doi: 10.1371/journal.pbio.3002704 (PMC11249258; doi:10.1371/journal.pbio.3002704)
Supplement: S10 Table — (DOCX) [file pbio.3002704.s010.docx]

**S10 Table. Primers for fine-mapping, amplification of *SfCHS2* genomic sequence and *SfCHS2* transcripts detection.**

| **Primer name** | **Primer sequence (5' to 3')** | **PCR product length (bp)** | **Application** |
| --- | --- | --- | --- |
| sf-5-S-1F | TGGAAGTGGAGAACCCTCCT | 1275 | Sanger sequencing of *SfCHS2* genomic sequence |
| sf-5-S-1R | GTTCCATGGCGTGTGGTGAT |  |  |
| sf-5-S-2F | CGTTCTTCTGGGTGATGCCT | 1370 |  |
| sf-5-S-2R | ATAATCCGGCATGGTGGTCT |  |  |
| sf-5-S-3F | CTCAGGCCACTGACTTTAGT | 1385 |  |
| sf-5-S-3R | ACCTGACGAAGCAAATGAGGAT |  |  |
| sf-5-S-4F | TAGGCACGCCGTAATAGCAT | 1428 |  |
| sf-5-S-4R | GCCCTTAGACACAACCGCAG |  |  |
| sf-cds-S-1F | TGTGAAGAAGGAGAGTGGGT | 1348 |  |
| sf-cds-S-1R | TCCCACCATCCGAGTGAGAT |  |  |
| sf-cds-S-2F | ATGTACTGGCTATCTCCGCT | 1192 |  |
| sf-cds-S-2R | CATGAACTTTGTAGAAGCGCT |  |  |
| sf-cds-S-3F | ACACACAGAGGGTGTTGTCT | 1547 |  |
| sf-cds-S-3R | ACCACGGCCTGTTGAAGAGT |  |  |
| sf-cds-S-4F | ATGTCGTGGATTTGGTTGCT | 963 |  |
| sf-cds-S-4R | TCAGGATCTACAATGCCCAAGT |  |  |
| sf-cds-S-5F | ATATGTCTGCGCTACGATGT | 1248 |  |
| sf-cds-S-5R | ACCATCTTTTCCTGTGTCGT |  |  |
| sf-cds-S-6F | CCATCTCACCAACGTGAGGT | 771 |  |
| sf-cds-S-6R | CCCACAGGATGGATGCGT |  |  |
| sf-cds-S-7F | TTTGGACGGCGACATTGACT | 1530 |  |
| sf-cds-S-7R | TCGAAGATGTTGGCGAGCGT |  |  |
| sf-cds-S-8F | TGGCTATGCACTTTGCTGCT | 1394 |  |
| sf-cds-S-8R | AGTTTTCTTCTGCGGCGTCT |  |  |
| sf-cds-S-9F | CGTCCAGTATGTTCACAGCT | 1432 |  |
| sf-cds-S-9R | TTCAGTGGAAATCTCGCTGT |  |  |
| sf-cds-S-10F | CCTGAGCCGACCCACCAG | 1439 |  |
| sf-cds-S-10R | GTCGTACTCCATGCTGGACT |  |  |
| sf-cds-S-11F | AACTCTCTGTTCGTGCTCGT | 1191 |  |
| sf-cds-S-11R | AGTCAATCTCCTCTTGGCGT |  |  |
| sf-cds-S-12F | ACGTCTGAACACCGACGACT | 1270 |  |
| sf-cds-S-12R | TCGAGATTCACTTTTCACGCGT |  |  |
| sf-cds-S-13F | TTGACACCACAAAGACCGAC | 1223 |  |
| sf-cds-S-13R | TGGTCACCAGTCGCAAATGT |  |  |
|  |  |  |  |
| 2992-SNP-F | AGTGCTAAAGAGGGCTAAGG | 296 | Fine-mapping |
| 2992-SNP-R | TCCAGGTAATCCGACACTGGG |  |  |
| 3106-SNP-F | AGGTCAGTGTCCCACTAC | 224 |  |
| 3106-SNP-R | ATGGCACTTCTACCGTTGG |  |  |
| 3136-SNP-F1 | ACTATAGATAGCTGGAGAGCC | 214 |  |
| 3136-SNP-R1 | ACGAAGCTAACACATTAGACG |  |  |
| 3105-SNP-F2 | AGCTCTTCCTCGCTAACCTC | 299 |  |
| 3105-SNP-R2 | TTTCTTCTGCGGCGTCTCTC |  |  |
| 3493-SNP-F | TGTTCGATGGAGGACATGGTC | 420 |  |
| 3493-SNP-R | TGTAAATGAAACCGCCAGCAG |  |  |
| 3409-SNP-F1 | AGATGGGAAGATGAGAGACCC | 263 |  |
| 3409-SNP-R1 | ACCTCTCCCTGATGAAAGAAG |  |  |
| 3178-SNP-F1 | TGTACCTGAAGTACCAGCTCC | 1078 |  |
| 3178-SNP-R1 | CTGTGGCTAGGTTCTACTTCC |  |  |
| 3547-SNP-F1 | AGCTATTGCAGAGCAGGAG | 175 |  |
| 3547-SNP-R1 | TACAAGTGCAATCGCGCTCTC |  |  |
|  |  |  |  |
| 11F7 | CGGTTTCGTGTTCCTCCTGT | 543 | *SfCHS2* transcripts detection |
| NeiR5 | GACCGCTGTGTACCACTTGT |  |  |
| NeiF2 | TCGGGTGGTAACAATACGGT | 878 |  |
| 11R4 | AGTCGTCGGTGTTCAGACGT |  |  |
| 11F7 | CGGTTTCGTGTTCCTCCTGT | 215 |  |
| 11R4 | AGTCGTCGGTGTTCAGACGT |  |  |
| 11 F2 | GGTGTTTGCGTTCGTGATGT | 121 |  |
| 10 R | GTCGTACTCCATGCTGGACT |  |  |
