# Supplementary material for: Retrotransposon-mediated disruption of a chitin synthase gene confers insect resistance to Bacillus thuringiensis Vip3Aa toxin
Source: PLoS Biol. 2024 Jul 2;22(7):e3002704. doi: 10.1371/journal.pbio.3002704 (PMC11249258; doi:10.1371/journal.pbio.3002704)

S1 Fig. Sample preparation strategy for bulked segregant analysis (BSA).

For resistance mapping, a single-pair cross was conducted between a male moth from the Sfru_R3 colony and a female moth from the SS colony to generate F1 progeny. The F1 progeny were raised on a normal diet, resulting in the production of F2 progeny. A total of 960 neonate larvae (480 for both selections) from the F2 generation were subjected to two different diets: a high Vip3Aa diet (4.0 μg/cm^2^) and a low Vip3Aa diet (0.1 μg/cm^2^) for a duration of five days. In the case of high Vip3Aa concentration, the individuals that developed into the 3rd instar after 5 days of exposure were classified as resistant to Vip3Aa (F2-R) (n=89). Conversely, for the low Vip3Aa concentration, the individuals that were still <3rd instar were considered susceptible to Vip3Aa (F2-S) (n=75). Following this classification, both the resistant and susceptible larvae from F2 generation were transferred to a normal diet until they reached the fifth instar stage.


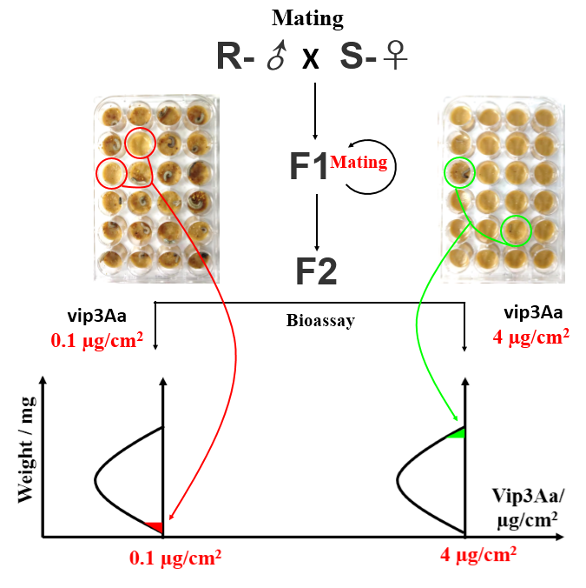

Supplement: S1 Fig — For resistance mapping, a single-pair cross was conducted between a male moth from the Sfru_R3 colony and a female moth from the SS colony to generate F1 progeny. The F1 progeny were raised on a normal diet, resulting in the production of F2 progeny. A total of 960 neonate larvae (480 for both selections) from the F2 generation were subjected to 2 different diets: a high Vip3Aa diet (4.0 μg/cm2) and a low Vip3Aa diet (0.1 μg/cm2) for a duration of 5 days. In the case of high Vip3Aa concentration, the individuals that developed into the third instar after 5 days of exposure were classified as resistant to Vip3Aa (F2-R) (n = 89). Conversely, for the low Vip3Aa concentration, the individuals that were still <third instar were considered susceptible to Vip3Aa (F2-S) (n = 75). Following this classification, both the resistant and susceptible larvae from F2 generation were transferred to a normal diet until they reached the fifth instar stage. (DOCX) [file pbio.3002704.s011.docx]
