# Supplementary material for: Retrotransposon-mediated disruption of a chitin synthase gene confers insect resistance to Bacillus thuringiensis Vip3Aa toxin
Source: PLoS Biol. 2024 Jul 2;22(7):e3002704. doi: 10.1371/journal.pbio.3002704 (PMC11249258; doi:10.1371/journal.pbio.3002704)
Supplement: S2 Fig — Neighbor-joining tree of Class A and Class B chitin synthases of insects. Class A enzymes make chitin for the exosekeleton; Class B enzymes make chitin for the peritrophic matrix. Both catalyze reversible elongation of the chitin chain (n to n+1) by addition of GlcNAc donated by UDP-GlcNAc: UDP-N-acetyl-D-glucosamine + [1,4-(N-acetyl-beta-D-glucosaminyl)]n ⇌ UDP + [1,4-(N-acetyl-beta-D-glucosaminyl)]n+1. Aedes aegypti: AaCHS1 = XP_021704891.1, AaCHS2 = XP_001651163.1. Drosophila melanogaster: DmCHS1 = AAG22215.3 (krotzkopf verkehrt), DmCHS2 = AAF51798.2. Manduca sexta: MsCHS1 = AAL38051.2, MsCHS2 = AAX20091.1. Tribolium castaneum: TcCHS1 = NP_001034491.1, TcCHS2 = NP_001034492.1. Spodoptera frugiperda: SfCHS1 = XP_050552783.1, SfCHS2 XP_050552796.1. Spodoptera litura: SlCHS1 = XP_022820392.1, SlCHS2 XP_050552796.1. The data underlying this figure can be found in S4 Data. (DOCX) [file pbio.3002704.s012.docx]

**S2 Fig. Insect chitin synthases.** Neighbor-joining tree of Class A and Class B chitin synthases of insects. Class A enzymes make chitin for the exosekeleton; Class B enzymes make chitin for the peritrophic matrix. Both catalyze reversible elongation of the chitin chain (n to n+1) by addition of GlcNAc donated by UDP-GlcNAc:

UDP-N-acetyl-D-glucosamine + [1,4-(N-acetyl-beta-D-glucosaminyl)]n ⇌ UDP + [1,4-(N-acetyl-beta-D-glucosaminyl)]n+1

*Aedes aegypti*: AaCHS1=XP_021704891.1, AaCHS2=XP_001651163.1. *Drosophila melanogaster*: DmCHS1=AAG22215.3 (krotzkopf verkehrt), DmCHS2=AAF51798.2. *Manduca sexta*: MsCHS1=AAL38051.2, MsCHS2=AAX20091.1. *Tribolium castaneum*: TcCHS1=NP_001034491.1, TcCHS2=NP_001034492.1. *Spodoptera frugiperda*: SfCHS1=XP_050552783.1, SfCHS2 XP_050552796.1. *Spodoptera litura*: SlCHS1=XP_022820392.1, SlCHS2 XP_050552796.1.
