# Supplementary material for: Retrotransposon-mediated disruption of a chitin synthase gene confers insect resistance to Bacillus thuringiensis Vip3Aa toxin
Source: PLoS Biol. 2024 Jul 2;22(7):e3002704. doi: 10.1371/journal.pbio.3002704 (PMC11249258; doi:10.1371/journal.pbio.3002704)
Supplement: S3 Fig — (A) By comparison to the wild-type sequence, it is possible to deduce the target site duplication (TSD) typically created by transposable element insertion, which is GAAGG in this case. This represents the last 4 bases of exon 21 and the first base of intron 21. (B) In the inserted allele, the LTRa immediately follows the first GAAGG, and since the first base of the LTRa is a T, a GT corresponding to the 5′-GU donor site of the pre-mRNA is created immediately after exon 21. The second GAAGG occurs immediately after the second LTR. (C) In accordance with the splicing rules, the entire insert could be spliced out, restoring the wild-type coding sequence (starting with the first G remaining from intron 21, through LTRa, Yaoer, LTRb and to the end of wild-type intron 22 with its 3′-AG acceptor site). (D) However, the inserted allele has the target site duplication GAAGG immediately after the second LTR, immediately preceding the T which is the second base of the wild-type intron 21. This restores the original 5′ donor splice site of intron 21. Therefore, if just the original intron 21 were spliced out, removing its 3′-AG acceptor site, this could block further splicing of the LTR retrotransposon from the pre-mRNA. In that case, read-through from exon 21 in the mature mRNA would encounter an in-frame stop codon in LTRa, leading to translation of a truncated protein of 1,355 residues, where the last 12 residues (VSILFTLLIYLL*) are encoded by the LTR. (E) Alternatively, splicing out just the entire Yaoer element, if possible, would result in another truncated protein ending in VS* due to read-through from exon 21 to intron 21. (DOCX) [file pbio.3002704.s013.docx]

**S3 Fig. Insertion site of LTR retrotransposon Yaoer and alternative splicing of SfCHS2.** (A) By comparison to the wild-type sequence, it is possible to deduce the target site duplication (TSD) typically created by transposable element insertion, which is GAAGG in this case. This represents the last four bases of exon 21 and the first base of intron 21. (B) In the inserted allele, the LTRa immediately follows the first GAAGG, and since the first base of the LTRa is a T, a GT corresponding to the 5'-GU donor site of the pre-mRNA is created immediately after exon 21. The second GAAGG occurs immediately after the second LTR. (C) In accordance with the splicing rules, the entire insert could be spliced out, restoring the wild-type coding sequence (starting with the first G remaining from intron 21, through LTRa, Yaoer, LTRb and to the end of wild-type intron 22 with its 3'-AG acceptor site). (D) However, the inserted allele has the target site duplication GAAGG immediately after the second LTR, immediately preceding the T which is the second base of the wild-type intron 21. This restores the original 5' donor splice site of intron 21. Therefore if just the original intron 21 were spliced out, removing its 3'-AG acceptor site, this could block further splicing of the LTR retrotransposon from the pre-mRNA. In that case, read-through from exon 21 in the mature mRNA would encounter an in-frame stop codon in LTRa, leading to translation of a truncated protein of 1355 residues, where the last 12 residues (VSILFTLLIYLL*) are encoded by the LTR. (E) Alternatively, splicing out just the entire Yaoer element, if possible, would result in another truncated protein ending in VS* due to read-through from exon 21 to intron 21.

(A) pre-insertion state

GTGAGTTAACTATCTTGA... TTTCCTTTTCGCAG

TGGTATTTCAGTAAGAAG CCGGACGACCTA

W Y F S K K P D D L

exon 21 intron 21 exon 22

(B) post-insertion state (TSD = target site duplication)

GTGTCTATTCTGTTTACATTACTTATTTATTTACTATGACAA...

TGGTATTTCAGTAAGAAG

W Y F S K K

exon 21 LTRa start

TSD

GTGAGTTAACTATCTTGA... TTTCCTTTTCGCAG

...TTGGGACGTGAACAGAAG CCGGACGACCTA

P D D L

LTRb end intron 21 exon 22

(C) splicing out the entire insert (GT- from LTRa, -AG from end of intron 21) yields the wild-type protein:

TGGTATTTCAGTAAGAAGCCGGACGACCTA ...

W Y F S K K P D D L

exon 21 exon 22

(D) splicing out just the original intron 21 (GT- from start of intron 21, -AG from end of intron 21) yields a truncated protein:

GTGTCTATTCTGTTTACATTACTTATTTATTTACTATGACAA...

TGGTATTTCAGTAAGAAG

W Y F S K K V S I L F T L L I V L L *

exon 21 LTRa start

(E) splicing out just the Yaoer element (GT- from LTRa, -AG from the TSD) also yields a truncated protein:

GTGAGTTAACTATCTTGA

TGGTATTTCAGTAAGAAG

W Y F S K K V S *

exon 21 intron 21
