# Supplementary material for: Retrotransposon-mediated disruption of a chitin synthase gene confers insect resistance to Bacillus thuringiensis Vip3Aa toxin
Source: PLoS Biol. 2024 Jul 2;22(7):e3002704. doi: 10.1371/journal.pbio.3002704 (PMC11249258; doi:10.1371/journal.pbio.3002704)
Supplement: S4 Fig — (A) Wild-type protein. (B) Mutant protein in Sfru_R3 alternatively spliced because of the Yaoer retrotransposon insertion. The C-terminal lumenal domain (C7) is truncated. (C) Knockouts generated by CRISPR/Cas9. The domain structure and numbers of residues in transmembrane domains and intervening loops were predicted using Phobius (https://phobius.sbc.su.se/). Transmembrane domains are numbered in red. (DOCX) [file pbio.3002704.s014.docx]

S4 Fig. Predicted structures of wild-type and mutant SfCHS2 proteins. (A) Wild-type protein. (B) Mutant protein in Sfru_R3 alternatively spliced because of the Yaoer retrotransposon insertion. The C-terminal lumenal domain (C7) is truncated. (C) Knockouts generated by CRISPR/Cas9.The domain structure and numbers of residues in transmembrane domains and intervening loops were predicted using Phobius (https://phobius.sbc.su.se/). Transmembrane domains are numbered in red.

**
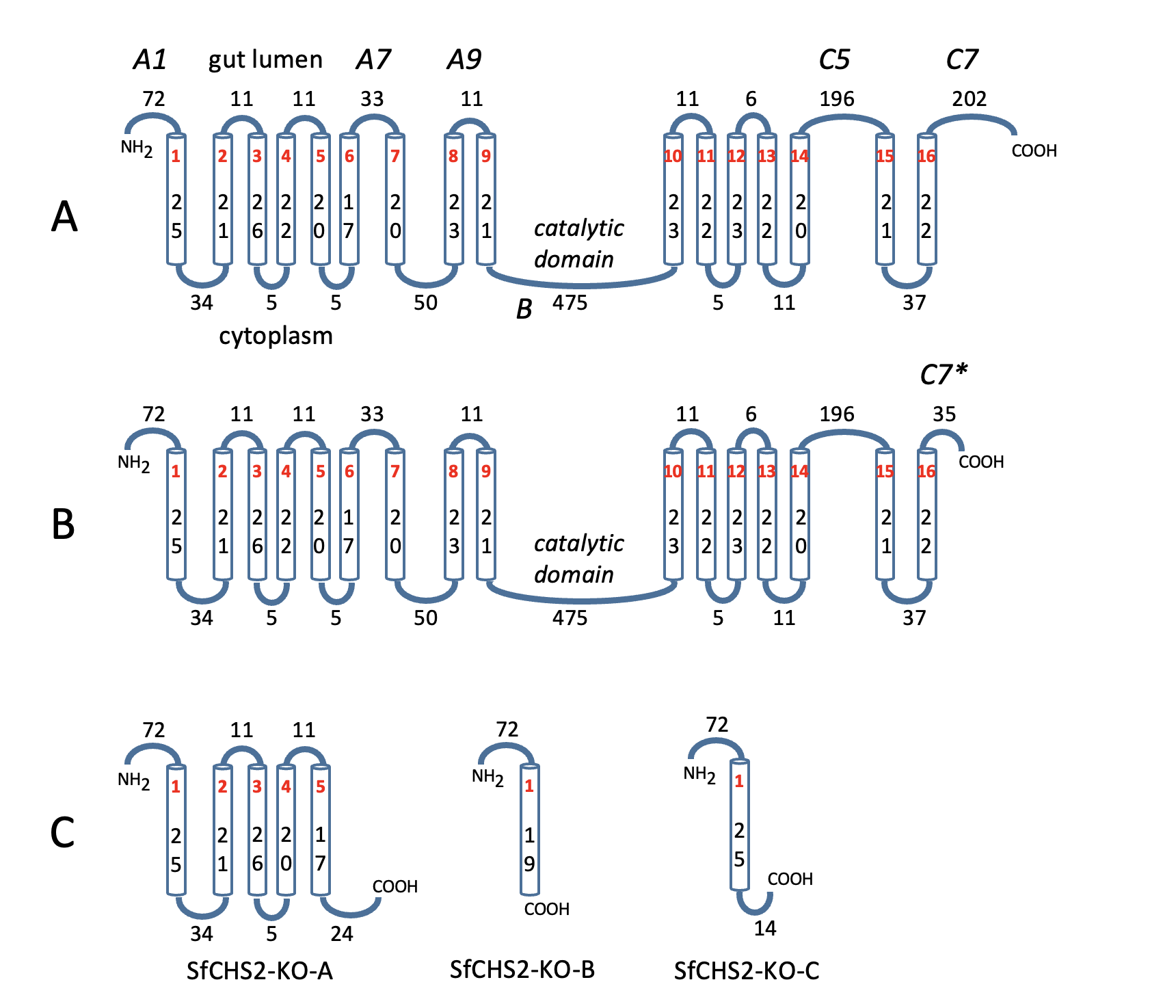
**
