# Supplementary material for: Retrotransposon-mediated disruption of a chitin synthase gene confers insect resistance to Bacillus thuringiensis Vip3Aa toxin
Source: PLoS Biol. 2024 Jul 2;22(7):e3002704. doi: 10.1371/journal.pbio.3002704 (PMC11249258; doi:10.1371/journal.pbio.3002704)
Supplement: S5 Fig — (A) SfCHS2 transcript detection via PCR and Sanger sequencing. Both wild-type and mutant SfCHS2 transcripts with Yaoer were detected in Sfru_R3. Furthermore, the transcripts including both Yaoer and intron 21 were detected in Sfru_R3 by amplification products generated by primer pairs of 11F7/ Nei R5 and Nei F2/11 R4. (B) SfCHS2 transcripts detection via Iso-Seq. In SS, multiple SfCHS2 transcripts without Yaoer were detected. In contrast, both SfCHS2 transcripts with and without Yaoer were detected in Sfru_R3. In addition, the transcripts of SfCHS2 that would have spliced out the Yaoer element while including the intron21 were undetected in Sfru_R3. The data underlying this figure can be found in S1 Raw Images. (DOCX) [file pbio.3002704.s015.docx]

**S5 Fig. Transcripts of *SfCHS2* in SS and Sfru_R3.** (A) *SfCHS2* transcript detection via PCR and Sanger sequencing. Both wild-type and mutant *SfCHS2* transcripts with Yaoer were detected in Sfru_R3. Furthermore, the transcripts including both Yaoer and intron 21 were detected in Sfru_R3 by amplification products generated by primer pairs of 11F7/ Nei R5 and Nei F2/11 R4. (B) *SfCHS2* transcripts detection via Iso-Seq. In SS, multiple *SfCHS2* transcripts without Yaoer were detected. In contrast, both *SfCHS2* transcripts with and without Yaoer were detected in Sfru_R3. In addition, the transcripts of *SfCHS2* that would have spliced out the Yaoer element while including the intron21 were undetected in Sfru_R3.
