# Supplementary material for: Retrotransposon-mediated disruption of a chitin synthase gene confers insect resistance to Bacillus thuringiensis Vip3Aa toxin
Source: PLoS Biol. 2024 Jul 2;22(7):e3002704. doi: 10.1371/journal.pbio.3002704 (PMC11249258; doi:10.1371/journal.pbio.3002704)

S6 Fig. Knockouts of *SfCHS2*. (A) SfCHS2-KO-B. (B) SfCHS2-KO-C. CRISPR/Cas9-mediated double sgRNA system and various types of mutations in G1 larvae identified through sequencing of individual PCR clones. Deleted bases are indicated as red dashes, and inserted bases are indicated as red letters. The CRISPR target sites and the number of deleted and inserted bases (+, insertion; –, deletion) are shown. The chromatogram shows the sequence of the mutant isolated from a homozygous knockout larva in G2.


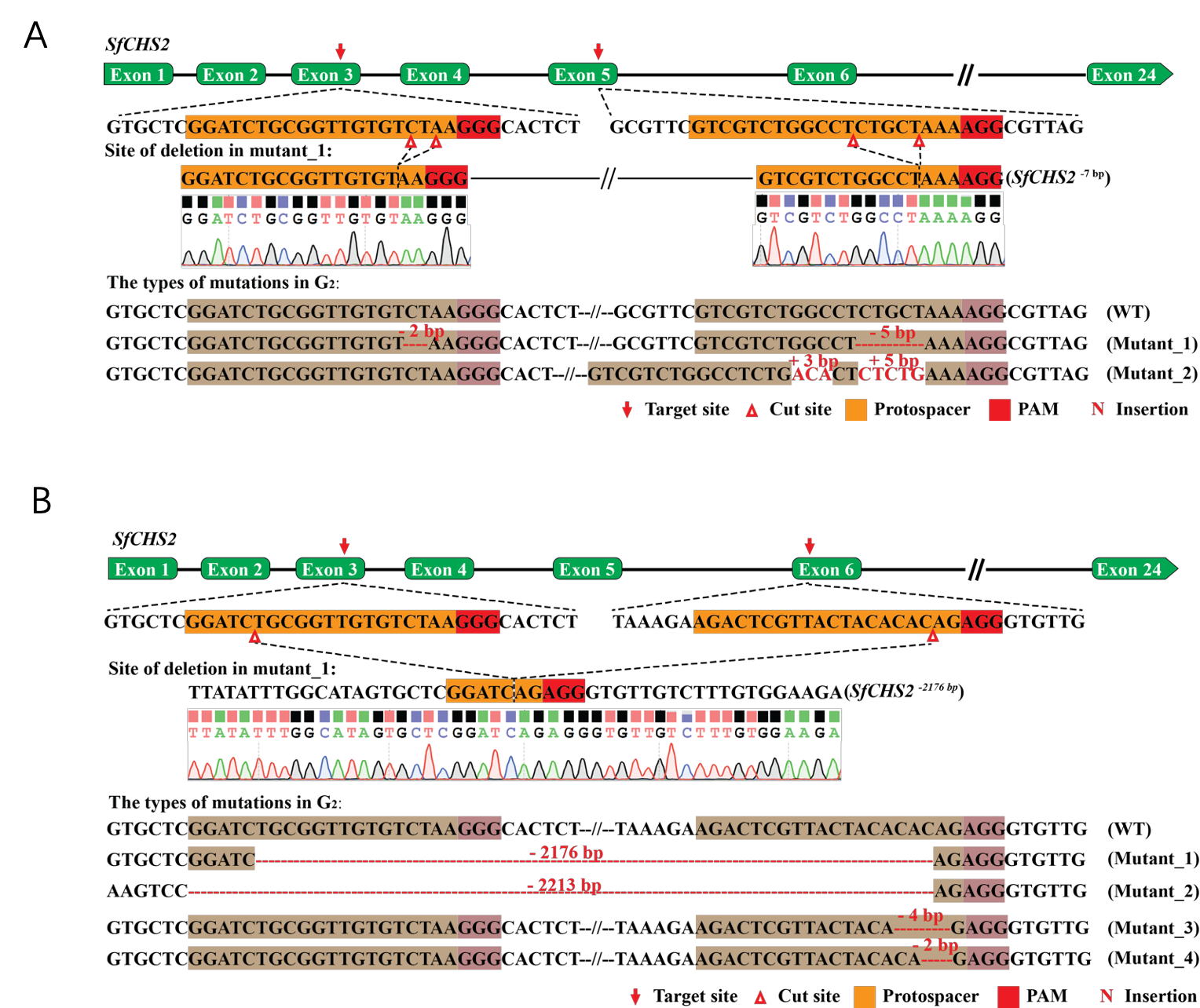

Supplement: S6 Fig — CRISPR/Cas9-mediated double sgRNA system and various types of mutations in G1 larvae identified through sequencing of individual PCR clones. Deleted bases are indicated as red dashes, and inserted bases are indicated as red letters. The CRISPR target sites and the number of deleted and inserted bases (+, insertion;–, deletion) are shown. The chromatogram shows the sequence of the mutant isolated from a homozygous knockout larva in G2. (DOCX) [file pbio.3002704.s016.docx]
