# Supplementary material for: Retrotransposon-mediated disruption of a chitin synthase gene confers insect resistance to Bacillus thuringiensis Vip3Aa toxin
Source: PLoS Biol. 2024 Jul 2;22(7):e3002704. doi: 10.1371/journal.pbio.3002704 (PMC11249258; doi:10.1371/journal.pbio.3002704)
Supplement: S8 Fig — A total of 540 S. frugiperda individuals collected from the field or laboratory were re-sequenced and mapped with the junctions of exon 21 or intron 21 and the Yaoer insertion (S9 Fig). One individual collected from the field of China in 2020 showed positive mapping. The red star shows the site for the positive mapping individual. The black circles show all other collection sites. See S9 Table for details of each sample. (DOCX) [file pbio.3002704.s018.docx]

**S8 Fig. Locations of field and laboratory populations of *S. frugiperda* screened for the Yaoer insertion in *SfCHS2.*** A total of 540 *S. frugiperda* individuals collected from the field or laboratory were re-sequenced and mapped with the junctions of exon 21 or intron 21 and the Yaoer insertion (S9 Fig). One individual collected from the field of China in 2020 showed positive mapping. The red star shows the site for the positive mapping individual. The black circles show all other collection sites. See Table S9 for details of each sample. The map was created using ggplot2 and base layer of the map was freely available from <https://www.gapminder.org/> under the CC-BY 4.0 license.

**
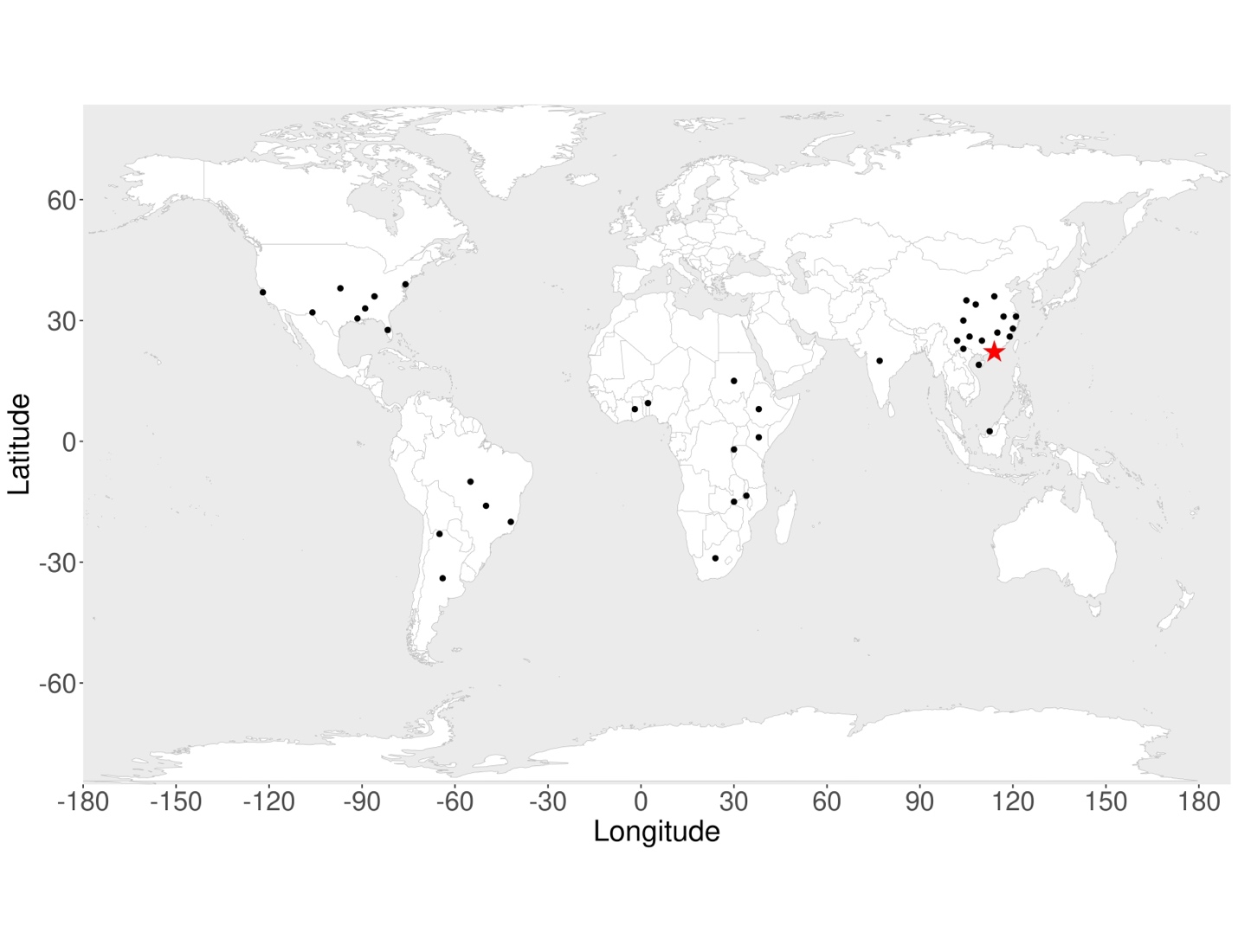
**
