# Supplementary material for: Retrotransposon-mediated disruption of a chitin synthase gene confers insect resistance to Bacillus thuringiensis Vip3Aa toxin
Source: PLoS Biol. 2024 Jul 2;22(7):e3002704. doi: 10.1371/journal.pbio.3002704 (PMC11249258; doi:10.1371/journal.pbio.3002704)
Supplement: S9 Fig — (A) Read coverage in the sample with the Yaoer insertion. (B) Read coverage in a wild-type sample. The insertion site sequences are depicted as colored bars. The reads from R2 are shown as gray horizontal bars and from R1 as horizontal white bars. Colored vertical bars within reads indicate variants. (DOCX) [file pbio.3002704.s019.docx]

S9 Fig. Detection of the Yaoer retrotransposon in *SfCHS2* in an individual collected from the field in China during 2020. (A) Read coverage in the sample with the Yaoer insertion. (B) Read coverage in a wild-type sample. The insertion site sequences are depicted as colored bars. The reads from R2 are shown as gray horizontal bars and from R1 as horizontal white bars. Colored vertical bars within reads indicate variants.

**
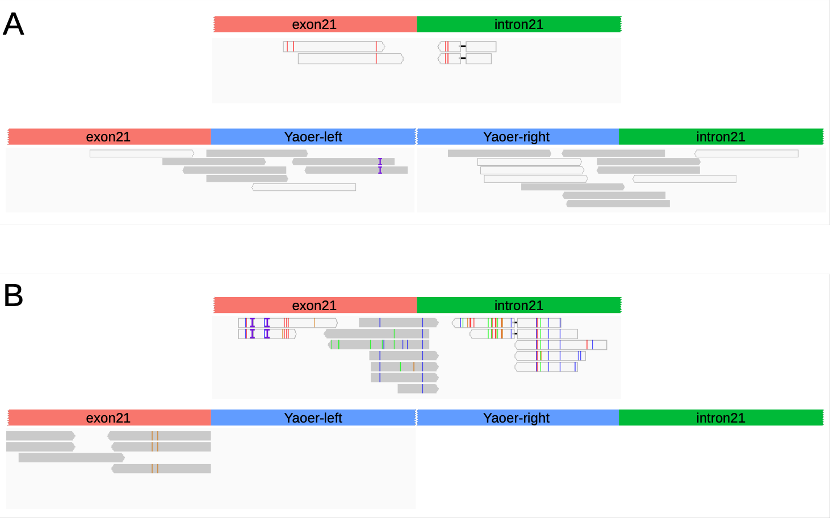
**
