# Supplementary material for: Retrotransposon-mediated disruption of a chitin synthase gene confers insect resistance to Bacillus thuringiensis Vip3Aa toxin
Source: PLoS Biol. 2024 Jul 2;22(7):e3002704. doi: 10.1371/journal.pbio.3002704 (PMC11249258; doi:10.1371/journal.pbio.3002704)
Supplement: S1 Raw Images — (PDF) [file pbio.3002704.s030.pdf]

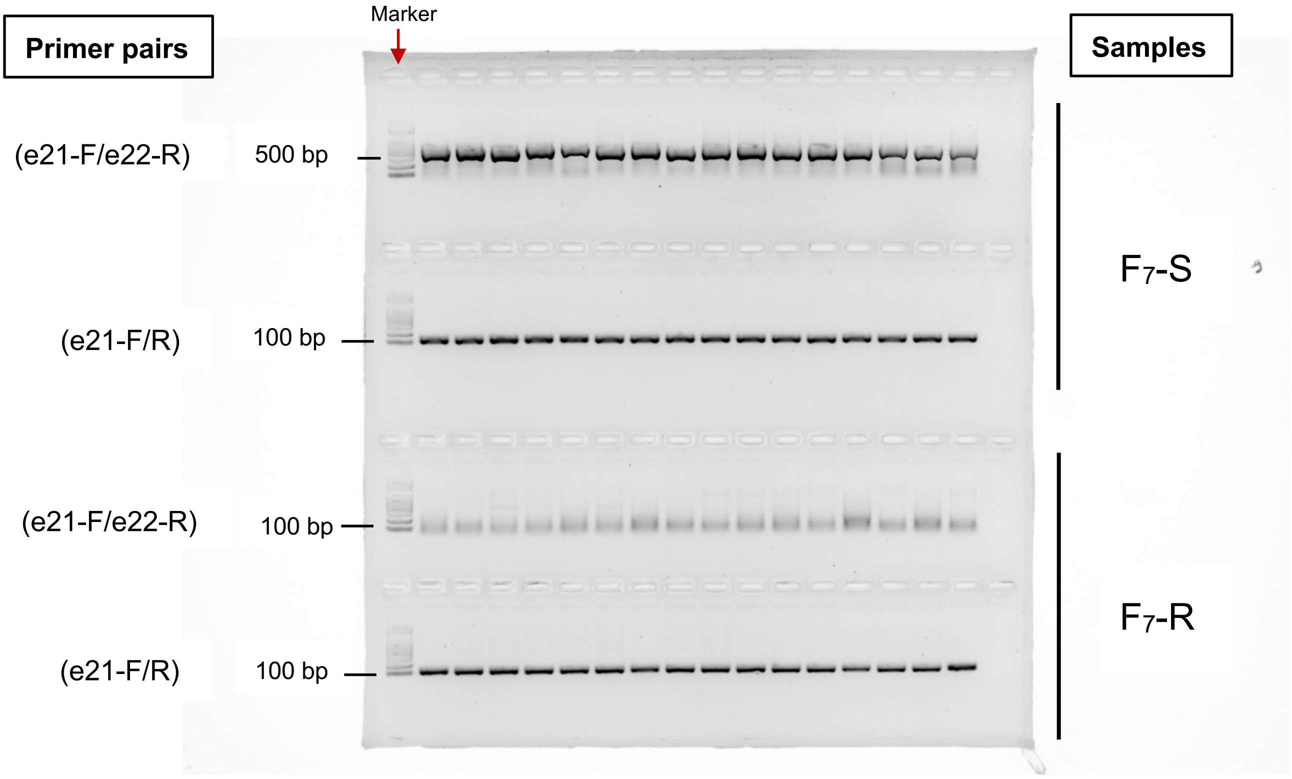

The **Figure 2C** in manuscript was originally generated from the gel image above. Two pairs of primer (e21-F/e22-R and e21-F/R) and the gDNA from F7-S (n=16) and F7-R (n=16) individual insect were used for PCR reaction. This image was captured by GelDoc Go Imaging System with Image Lab Touch Software (Bio-Rad). To make it more clear, the dark and light areas of this image was inverted suitably.

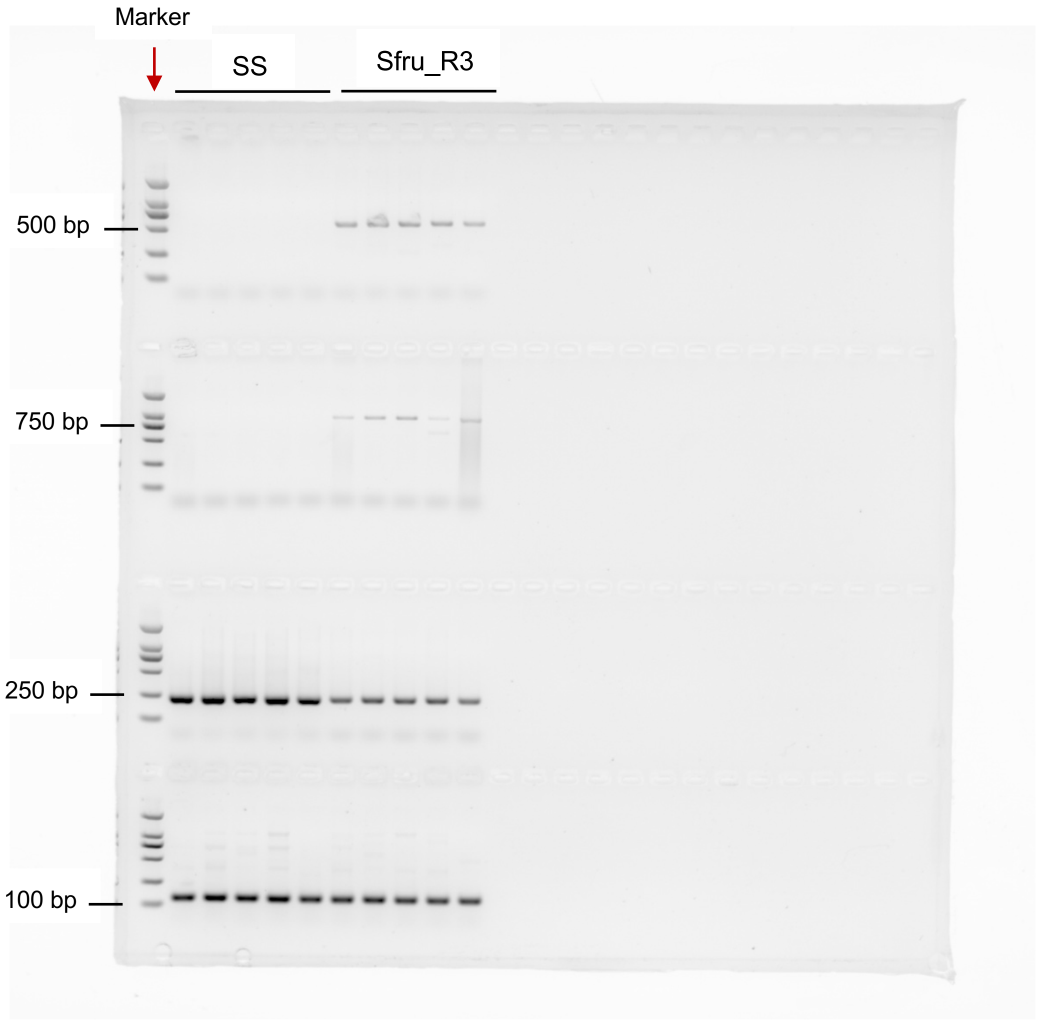

The **Figure S5A** in Supporting information was originally generated from the gel image above. A total of four pairs of primer (11 F7/Nei R5, Nei F2/11 R4, 11 F7/11 R4 and 11 F2/10 R) and the cDNA from SS (n=5) and Sfru_R3 (n=5) were used for PCR reaction. This image was captured by GelDoc Go Imaging System with Image Lab Touch Software (Bio-Rad). To make it more clear, the dark and light areas of this image was inverted suitably.
