# Supplementary material for: Retrotransposon-mediated disruption of a chitin synthase gene confers insect resistance to Bacillus thuringiensis Vip3Aa toxin
Source: PLoS Biol. 2024 Jul 2;22(7):e3002704. doi: 10.1371/journal.pbio.3002704 (PMC11249258; doi:10.1371/journal.pbio.3002704)
Supplement: Attachment — Submitted filename: Response to Reviewers comments PLoS Biology 5-13-24.docx [file pbio.3002704.s031.docx]

Point-by-Point Response to Reviewers’ Comments PBIOLOGY-D-24-00644R1

Reviewer #1: 
This manuscript reports the finding that chitin synthase 2 (CHS2) mutation confers high level resistance to Bt toxin Vip3Aa in insects. The authors obtained a strain of Spodoptera frugiperda, Sfru_R3, highly resistant to Vip3Aa (resistance ratio=5560) by selecting a susceptible colony with Vip3Aa for 17 generations. Using BSA (bulk segregant analysis) and Amp-seq based mapping, the resistance was mapped to the SfCHS2 locus. By DNA sequencing of the genes and RNA-seq to identify the midgut-expressed genes in locus, the authors identified the resistance associated mutation in the SfCHS2. The mutation was an insertion of a transposon in the SfCHS2 gene. The high level resistance to Vip3Aa was reproducible by introducing knockout mutations in the CHS2 gene in S. frugiperda and also in two other insects, S. litura and M. separata. The finding of an important role of CHS2 in toxicity of a Bt toxin in insects is new to the field of Bt research. The techniques and experimental designs in this study to identify the resistance conferring gene mutation are sound.

REPLY: We thank the reviewer for the positive feedback.

Other comments: 
1.    It is interesting to note that in a recent report from this same group a moderate level Vip3Aa resistant strain was selected from the very same susceptible colony (DH-S) by selection with Vip3Aa and the genetic mechanism for resistance is independent of CHS2 mutation. As Bt resistance development in insect populations in agriculture is a process of selection of resistant alleles. Even though the selection in this study is in lab, it would be interesting to readers, if authors could provide any insights to the observations that two similar selections from the same susceptible colony resulted in two very different resistant strains with different genetic mechanisms and the two mechanisms were not co-selected in a strain.

REPLY: Revised as suggested by expanding insights in the Discussion from this observation to include the underlined sentence:

“It is striking that the genetic basis of resistance differs between the two Vip3Aa-resistant strains derived by laboratory selection from the SS strain, which was started with approximately 100 *S. frugiperda* collected from field in 2019 [31]. This suggests that each of the two subsets of the SS strain selected in the laboratory with Vip3Aa to obtain a resistant strain contained one of the two different resistance alleles identified but not the other. Thus, field-evolved resistance to Vip3Aa in this pest, which would arise from selection of a much larger and more genetically diverse population than the SS strain, could entail a diverse genetic basis of resistance as seen with resistance to Cry1Ac in *Helicoverpa armigera* [56]. Accordingly, resistance to Vip3Aa in a single population might be conferred by mutations affecting one gene in some individuals and mutations affecting other genes in other individuals.”

2.    Another interesting observation from the selection process is that the resistance trait selected in this study was very high level resistance controlled by a single gene mutation, but the resistance level appeared to increase rather slowly in the selection process. As the dominance H is 0 at the dose 2 μg Vip3Aa per cm^2^, with the selection doses (all > 2) all or close to all heterozygous larvae would be killed by Vip3Aa in each round of selection. One would expect the resistance level would have increased rather quickly by selection with Vip3Aa, as in theory only homozygous highly resistant larvae would survive the selection. However, after one year of selections with the toxin, the resistance level was only 7.4-fold. The resistance level increased rapidly only after 2021.05. Does this observation suggest the CHS2 gene mutation occurred in the lab in the selection process at one point during that time?

REPLY: Revised to address this question by adding the following paragraph to the Discussion:

“Our finding of one individual homozygous for the Yaoer insertion and no heterozygotes in the 158 field-collected insects screened from China yields an estimated frequency of 0.00633 for this mutation in China. Based on this estimate, the probability of at least one copy of the Yaoer insertion in the approximately 100 field-sampled individuals from China used to start the SS strain is approximately 0.72 (1 – [1-0.00633]^200^). Thus, it is likely that at least one copy of the Yaoer insertion occurred in the field-collected individuals that started the SS strain, even though we cannot exclude the possibility that this mutation arose in the SS strain after it was established in the lab. The initial slow response of Sfru_R3 to selection with Vip3Aa followed by an exponential increase in the resistance ratio (Table S1) is similar to the general pattern expected for the response to selection conferred by a recessive resistance allele that is rare initially (57).”

3.    In Fig 1, please explain in legend what the solid line indicates (mean of deltaSNP index in 1 kb window?).

REPLY: Revised as suggested by adding this to the legend for Fig. 1B: “The solid line indicates the LOESS regression fitted ΔSNP-index value.” Loess regression is a nonparametric technique that uses local weighted regression to fit a smooth curve through points in a scatter plot.

4.    In line 142 "Results from Sanger sequencing of the full-length genomic sequences of SfCHS2 …": Methods for this experiment are missing the manuscript.

REPLY: Revised as suggested. We added the following information to the Methods (L502-518):

**“Sanger sequencing of the *SfCHS2* genomic sequence in SS and Sfru_R3**

For genomic sequencing of *SfCHS2* in SS and Sfru_R3, a total of 17 pairs of primers (Table S10) across the 5’ UTR and CDS region of *SfCHS2* were designed on the basis of the genomic sequence of *SfCHS2* from the reference genome assembly (AGI-APGP_CSIRO_Sfru_2.0, GCF_023101765.2). The PCR products were directly Sanger sequenced (Sangon Biotech Co., Ltd. Shanghai, China). To assure the accuracy of the full-length genomic sequence of SfCHS2, primers for the overlapping region with a range of 100-500 bp between each two adjacent PCR products were designed. All the primers for amplification of *SfCHS2* genomic sequence were designed based on the exon sequence. The length of all the PCR products ranged from 770 to 1550 bp. The gDNA from SS and Sfru_R3 were isolated as described above. PCR reactions were performed in a S1000 Thermal Cycler (Bio-Rad) using the 2×Taq Plus Master Mix II (Vazyme, Nanjing, China) as follows: initial denaturation 95 °C for 3 min, followed by 35 cycles of 95 °C for 15 s, 55°C for 15 s and 72 °C for 30-60 s, and a final extension of 5 min at 72 °C. For the amplification of the genomic sequence across intron 21 of SfCHS2 in Sfru_R3, the extension time was lengthened to 3, 5, and 10 min, since no PCR products were obtained for Sfru_R3 under a shorter extension time. The PCR products (~7 kb) between exon 21 and exon 22 from Sfru_R3 were also directly Sanger sequenced as described above.”

5.    The sentence from line 214-216 does not tell much significant, as the important event is mutation of CHS2 causing resistance to Vip3Aa and transposition of a transposon is not likely gene specific for Vip resistance or Cry resistance.

REPLY: The sentence is: “Whereas previous findings show that transposable elements can cause resistance to Cry toxins^45-50^, the results here are the first to identify this mechanism in resistance to Vip3Aa.”

Thus, this sentence indicates that the specific transposon insertion we found causes resistance to Vip3Aa. It does not claim that generally the insertion of a transposon is gene specific for Vip resistance or Cry resistance.

6.    The conclusive sentence from line 225 to 227 "The finding … suggests that this domain plays a key role in susceptibility to Vip3Aa": I would suggest remove this sentence, as there is no evidence that the truncated protein remains to be a membrane protein in the midgut as the wild type.

REPLY: If the truncated protein (which lacks the C7 domain) does not remain a membrane protein in the midgut and this change causes resistance, this would confirm our statement that this domain plays a key role in susceptibility.

7.    Line 247 to 249, authors stated the finding of "Yaoer" from the SS strain "originated in Ruili City". Please provide a citation or the result in this manuscript.

REPLY: The statement indicates that we found Yaoer in the SfRu_R3 strain and that the SS strain (not Yaoer) originated from Ruili City. We revised for clarity:

“We found the Yaoer retrotransposon in the *SfCHS2* gene in the SfRu_R3 strain that was derived from the SS strain that originated in Ruili City in southwestern China and in a field-collected individual from Jiangmen City in southeastern China, 1500 km from Ruili City.”

8.    In Methods, numerous biocomputing software packages were used, but no references were provided for the software. Please add reference or sources for these software in Methods.

REPLY: Revised as suggested. We added the references for all software in the Methods.

---------------------------------------------------------------- 
Reviewer #2: 

In this manuscript by Liu and colleagues a compelling case is made that very high levels of resistance to the insecticidal protein VIP3A are generated by mutations in a chitin synthase gene of pest moths. The story starts with 17 generations of  lab selection on the fall army worm (Spodoptera frugiperda; a major invasive pest of agriculture) to ever increasing doses of VIP3a, which is a bacterial derived toxin that has a different (but poorly characterised) mode of action than Bt Cry toxins. Genetic mapping shows that gene is autosomal, its dominance depends on the dose, and is located on chromosome 1. Then bulked segregant analysis is used to map the gene at a fine scale and with some sensible elimination of candidates based on their expression in the midgut (where these toxin act) and to proteins that are membrane bound  the authors are lead to a chitin synthase thought to have a role in generating the peritrophic membrane (that is sloughed off contributing to bolus 
during digestion). The putative causal mutation is a Transposable element (they named Yaoer) that happens to have inserted within the splice donor of intron 21. CRISPR experiments in the fall army worm and two other species irrefutably confirm the role of this gene as mutations that must inactivate the gene also give VIP3A resistance. 

This represents a very significant advance in the field as VIP3A resistance has not been characterised to gene-resolution before and the finding will shape the way that field resistance is interpreted and screened for by researchers from now on.   

The experimental logic that unfolds throughout the paper makes it an enjoyable read.

REPLY: We thank the reviewer for the positive feedback.

I do however have a few comments and questions. 

1. The resistance variant would have been present in the moths that were used to start the lab colony. It did not arise via mutation in the lab colony because the same mutation has been found in 1/540 moths collected independently. If samples had been kept it'd be interesting to retrospectively track its frequency through the 17 generations of selection.  Regardless the methods should state the approximate size of the original lab population.

REPLY: Revised as suggested by inserting this in Methods: “Approximately 100 field-collected individuals were reared to pupae on non-Bt corn in the laboratory to start the strain [31].”

We agree it would be interesting to retrospectively track the frequency of the mutation but we do not have the samples to do that.

2. The paragraphs initial describing the transposon would benefit from more attention. In particular it seems to me that 3' Rapid Amplification of cDNA Ends (RACE) would give a more satisfactory understanding of the transcriptional consequences of the insertion - rather than the hypothetical possibilities articulated in FigS3. Perhaps this is what ISO-Seq does - but if so either be more explicit or provide a method citation. And the text about the 5' splice site being downstream from Yaoer is very confusing. Also line 142 refers to Sanger sequencing of a PCR product of at least 6427bp- I don't think the implied cloning is mentioned in the methods.

REPLY: Indeed, Iso-Seq does exactly that--it directly sequences full-length transcripts. The Methods for Iso-Seq are provided in more detail in the revision:

“For Iso-Seq, the total RNA of midguts from 5th-instar larvae of SS and Sfru_R3 were isolated and sent to BerryGenomics (Beijing, China) for library construction and sequencing. For each sample, 200 ng of total RNA was input for cDNA synthesis and amplification using NEB Next Single Cell/Low Input cDNA Synthesis & Amplification Module (New England BioLabs Inc., Ipswich, MA, USA) following the manufacturer’s standard protocol. A total of 13 PCR cycles were used to generate sufficient quantities of cDNA for PacBio Iso-Seq library preparations. SMRTbell Iso-Seq libraries was constructed using Express Template Prep 2.0 (Pacific Biosciences, Menlo Park, CA, USA) by following the manufacturer’s Iso-Seq Express Template Preparation protocol. The SS and Sfru-R3 Iso-Seq libraries were run on a single Sequel system SMRT Cell using sequencing chemistry 3.0 with 4-hour pre-extension and 20-hour movie time. Raw reads were processed into circular consensus sequence (CCS) reads as per the manufacturer’s standard pipeline (SMRT Link version 8.1).”

The new Figure S5, part B shows the Iso-Seq results. This figure is slightly corrected from the previous version. Iso-Seq from Sfru_R3 shows transcripts that terminate within the Yaoer element, partly eliminating the C7 region (Sfru_R3, transcript 02, transcript 03). In these transcripts, the Yaoer element was not spliced out, and transcription terminated within the Yaoer element. Figure S5 also shows transcripts in which the entire Yaoer element plus intron 21 was spliced out (Sfru_R3, transcript 01, transcript 04). The result is a wild-type transcript encoding the entire protein. Neither transcript is expected to be subjected to nonsense-mediated decay.

The methods for Sanger sequencing of the *SfCHS2* genomic sequence in SS and Sfru_R3 including the Yaoer sequence have been added to the Methods, as noted above in the response to comment 4 of Reviewer 1.

3. It is interesting that the Yaoer element does not appear to disrupt the whole transcript but just the end of the transcript (text and figure 2C). This suggests that nonsense mediated decay does not occur. The authors note that the truncated protein may have some residual function. But what of the CRISPR knockouts that occur in exon 5 to 6. Presumably the transcripts produced from that are produced from them are not stable and yet homozygotes are viable. Is the fitness cost of the CRISPR knockouts less than the fitness cost of the Yaoer bearing alleles? (the figure legend of figure 2 does not state whether section c is from gDNA or cDNA nor are the molecular markers used stated).

REPLY: Indeed, nonsense-mediated decay is not expected to occur for the transcript that terminates within Yaoer (Fig S5B), because there are no exon-exon junctions following the stop codon. Nonsense-mediated decay might occur for the transcripts in the CRISPR knockouts in exon 5 to 6, but homozygotes are viable in the lab despite complete absence of the peritrophic matrix. As reported in the Results, we did not detect a fitness cost associated with the alleles bearing the Yaoer insertion. We did not evaluate the fitness cost on maize in the CRISPR knockouts, but it could be substantial and might depend on whether larvae are fed artificial diet or plant material. gDNA was used for *SfCHS2* genotype identification and it has been stated in the legend of Figure 2.

4. I note a phylogenetic tree of the chitin synthase gene is provided in the sups. Personally I'd prefer a figure of the enzymatic reaction that the chitin synthase performs. Perhaps that could be added as well?

REPLY: Revised as suggested. A description of the enzymatic reaction has been added to the caption of Fig. S2:

“Neighbor-joining tree of Class A and Class B chitin synthases of insects. Class A enzymes make chitin for the exosekeleton; Class B enzymes make chitin for the peritrophic matrix. Both catalyze reversible elongation of the chitin chain (n to n+1) by addition of GlcNAc donated by UDP-GlcNAc:

UDP-N-acetyl-D-glucosamine + [1,4-(N-acetyl-beta-D-glucosaminyl)]^n^ ⇌ UDP + [1,4-(N-acetyl-beta-D-glucosaminyl)]^n+1^”

5. line 90 refers to non-Bt maize , and by implication this suggests that VIP3Aa is from Bt (even though it is not a Cry protein) which is no doubt obvious to authors but rewording would make it clearer to more generalist readers.

REPLY: Revised as suggested to “On non-transgenic maize plants”.
